# Plasma antibodies from humans infected with zoonotic simian foamy virus do not inhibit cell-to-cell transmission of the virus despite binding to the surface of infected cells

**DOI:** 10.1371/journal.ppat.1010470

**Published:** 2022-05-23

**Authors:** Mathilde Couteaudier, Thomas Montange, Richard Njouom, Chanceline Bilounga-Ndongo, Antoine Gessain, Florence Buseyne

**Affiliations:** 1 Institut Pasteur, Université Paris Cité, CNRS UMR3569, Unité d’Epidémiologie et Physiopathologie des Virus Oncogènes, Paris, France; 2 Centre Pasteur du Cameroun, Yaoundé, Cameroon; 3 Ministère de la Santé Publique, Yaoundé, Cameroon; Imperial College London, UNITED KINGDOM

## Abstract

Zoonotic simian foamy viruses (SFV) establish lifelong infection in their human hosts. Despite repeated transmission of SFV from nonhuman primates to humans, neither transmission between human hosts nor severe clinical manifestations have been reported. We aim to study the immune responses elicited by chronic infection with this retrovirus and previously reported that SFV-infected individuals generate potent neutralizing antibodies that block cell infection by viral particles. Here, we assessed whether human plasma antibodies block SFV cell-to-cell transmission and present the first description of cell-to-cell spreading of zoonotic gorilla SFV. We set-up a microtitration assay to quantify the ability of plasma samples from 20 Central African individuals infected with gorilla SFV and 9 uninfected controls to block cell-associated transmission of zoonotic gorilla SFV strains. We used flow-based cell cytometry and fluorescence microscopy to study envelope protein (Env) localization and the capacity of plasma antibodies to bind to infected cells. We visualized the cell-to-cell spread of SFV by real-time live imaging of a GFP-expressing prototype foamy virus (CI-PFV) strain. None of the samples neutralized cell-associated SFV infection, despite the inhibition of cell-free virus. We detected gorilla SFV Env in the perinuclear region, cytoplasmic vesicles and at the cell surface. We found that plasma antibodies bind to Env located at the surface of cells infected with primary gorilla SFV strains. Extracellular labeling of SFV proteins by human plasma samples showed patchy staining at the base of the cell and dense continuous staining at the cell apex, as well as staining in the intercellular connections that formed when previously connected cells separated from each other. In conclusion, SFV-specific antibodies from infected humans do not block cell-to-cell transmission, at least *in vitro*, despite their capacity to bind to the surface of infected cells.

**Trial registration**: Clinical trial registration: www.clinicaltrials.gov, https://clinicaltrials.gov/ct2/show/NCT03225794/.

## Author summary

Foamy viruses are the oldest known retroviruses and have been mostly described to be nonpathogenic in their natural animal hosts. Simian foamy viruses (SFVs) can be transmitted to humans, in whom they establish persistent infection, as have the simian viruses that led to the emergence of two major human pathogens, human immunodeficiency virus type 1 (HIV-1) and human T lymphotropic virus type 1 (HTLV-1). Such cross-species transmission of SFV is ongoing in many parts of the world where humans have contact with nonhuman primates. We previously showed high titers of neutralizing antibodies in the plasma of most SFV-infected individuals. These antiviral antibodies can inhibit cell-free virus entry. However, SFV efficiently spread from one cell to another. Here, we demonstrate that plasma antibodies do not block such cell-to-cell transmission, despite their capacity to bind to the surface of infected cells. In addition, we document for the first time the cell-to-cell spread of primary zoonotic gorilla SFV.

## Introduction

Foamy viruses (FVs) are complex retroviruses that are widespread among several mammalian species, including nonhuman primates (NHPs) [1]. Cross-species transmission of simian FV (SFV) to humans occurs mostly through bites [2,3]. SFV from apes and Old World monkeys establish lifelong infection, with the persistence of replication-competent viruses in the human host [4]. FV infection is mostly asymptomatic in natural and accidental hosts but associated with subclinical hematological and kidney alterations, as well as persistent immune activation in humans and cats [5–8]. Human infection with zoonotic SFV is an original natural model for the study of viral emergence from a simian reservoir. Humans have frequent contacts with NHPs in Africa, Asia and Central and South America, occurring in various contexts, such as hunting bushmeat, visiting religious sites, occupational exposure, owning pets and protecting crops [9–11]. The rate of simian-to-human transmission is higher for SFV than simian immunodeficiency viruses (SIV) and simian T-cell leukemia viruses (STLV), the two retroviruses that have emerged in the human population [12,13]. Despite repeated transmission of SFV to humans and their lifelong persistence in their novel hosts, these viruses have not been further transmitted between humans [2,3]. Therefore, SFV infection is probably efficiently contained by the innate and adaptive immune responses of infected humans.

Antibody-mediated antiviral activity restricts viral spread in the host, as well as viral transmission between hosts. FV-specific neutralizing antibodies have been detected in felines, NHPs and humans [14–17]. They target a variant region of the surface domain that overlaps with the receptor binding site [17,18]. The variant region is dimorphic and two envelope (*env)* genotypes cocirculate in simian and feline populations [19–22]. Aside from genotype-specific neutralizing antibodies, FV-infected hosts produce envelope protein (Env)-specific antibodies that bind to the leader peptide (LP) and transmembrane (TM) Env domains [23,24].

FVs spread through infection of susceptible cells by cell-free virions and cell-to-cell transmission. Cell-associated spread is the dominant or exclusive mode of transmission for several strains, including bovine FVs (BFVs) [25,26] and certain gorilla SFVs [27]. In BFV, the C-term cytoplasmic domain of Env is a major determinant of the cell-free and cell-associated transmission routes [28,29]. Heparan sulfate is an attachment factor for FVs [30,31] for which the receptor is unknown. Cell-free FVs enter cells through endocytosis and the fusion of viral and cellular membranes occurs at low pH, except for the laboratory-adapted prototype foamy virus (CI-PFV) strain [32,33]. Cell-free FV transmission is supported by the intracellular budding of FV particles and their release after cytopathic cell lysis. FV Env carries an endoplasmic reticulum retention signal in the cytoplasmic LP and TM domains [34,35]. The intracytoplasmic Env LP subdomain binds to Gag. This interaction relieves Env retention in the ER and targets FV capsids to intracellular budding sites. Concomitant to intracellular particle formation, budding has been observed at the plasma membrane [25,36–38]. To date, the generation of multinuclear syncytia resulting from Env-mediated fusion of infected cells is the only FV cell-to-cell transmission route to have been described [39].

The capacity of FV-specific antibodies to block cell-to-cell transmission has been addressed in a single report, in which neutralizing SFV-immune rabbit sera neutralizing cell-free SFV did not block cell-to-cell spread *in vitro* [40]. Here, we addressed this question by conducting a novel study using human plasma samples from infected Central-African hunters with well-characterized neutralizing activity against cell-free zoonotic gorilla SFV strains that have undergone limited *in vitro* passage [17,41]. We show that human immune plasma does not block the cell-to-cell spread of SFV. Env expression at the cell surface is thought to mediate cell-to cell transmission of the virus. Env from the zoonotic gorilla SFV strains was localized to the plasma membrane and in intracellular structures. SFV spreads between cells through fusion and contact without fusion. Plasma antibodies bind to the surface of SFV-infected cells, raising the possibility that they may limit viral spread *in vivo* by non-neutralizing activity.

## Materials and methods

### Ethics statement

Participants gave written informed consent. Ethics approval was obtained from the relevant national authorities in Cameroon (the Ministry of Health and the National Ethics Committee) and France (Commission Nationale de l’Informatique et des Libertés, Comité de Protection des Personnes Ile de France IV). The study was registered at www.clinicaltrials.gov, https://clinicaltrials.gov/ct2/show/NCT03225794/.

### Human plasma samples

Blood samples were drawn from adult populations living in villages and settlements across the rainforest in Cameroon or Gabon. SFV infection was diagnosed by a clearly positive Gag doublet on western blots using plasma from the participants and the amplification of the integrase gene and/or LTR DNA fragments by PCR using cellular DNA isolated from blood buffy-coats [42]. The origin of the SFV was identified by phylogenetic analysis of the sequence of the integrase gene [42]. Plasma samples from 29 participants were used for this study (S1 Table). Nine participants were not infected with SFV and 20 were infected with a gorilla SFV. Their neutralization profile and genotype-specific PCR were performed as described [17].

### Cells, viruses and plasmids

BHK-21 (ATCC-CLL-10, hamster kidney fibroblast) cells were cultivated in DMEM supplemented with 5% fetal bovine serum (FBS, PAA Laboratories) and HT1080 cells (ECACC 85111505, human fibrosarcoma) in Eagle’s Minimum Essential Medium with Earle’s Balanced Salts and L-glutamine (EMEM-EBSS, Lonza) supplemented with 10% FBS+ 1% L-glutamine (Gibco). Human embryonic kidney 293T cells (Cat. N° 12022001, Sigma) were cultured in DMEM-10% FBS. We used two zoonotic gorilla SFV strains, SFVggo_huBAD468 (GI-D468) and SFVggo_huBAK74 (GII-K74), isolated from Cameroonian hunters, with limited *in vitro* passage [41] and the laboratory adapted CI-PFV derived from SFVpsc_huHSRV13 and SFVpve_Pan2 (CII-SFV7) [43]. Viral stocks were produced in BHK-21 cells and titrated with gorilla foamy virus activated β-galactosidase (GFAB) indicator cells [44]. The CI-PFV-GFP molecular clone was derived the HSVR13 molecular clone that encodes infectious CI-PFV [45,46]. The 5’ U3 region was replaced by the immediate/early CMV promoter to obtain Tas-independent constitutive expression (pc13 plasmid, [47]). We inserted the human codon-optimized sequence of *gfp* 5’ of LP, as previously done for FV vectors [33]. The *env* and *pol* genes overlap by 53 nucleotides. We inactivated the 3 ATG sites 5’ of *env* by nucleotide substitution, resulting in a silent mutation in the *pol* coding sequence. We inserted the *gfp* gene after the *pol* stop codon fused to the full *env* gene, in which we introduced silent mutations over the first 53 nucleotides to avoid homologous recombination. Infectious CI-PFV-GFP was produced by transfecting 293T cells with 2.3 μg plasmid DNA per 5.10^5^ cells in the presence of LipoD293 *In Vitro* DNA Transfection Reagent (Signagen Laboratories) at a 3:1 ratio. After 48 h of culture, supernatants were harvested, filtered and used to infect BHK-21 cells. The cells were passed twice a week. Once the cytopathic effect (CPE) was visible, uninfected BHK-21 cells were added at each passage (2:1) to propagate the virus. GFP expression was stable over five months of culture.

We generated the sENV-GFP plasmid encoding GI-D468 fused to GFP in C-term in which we mutated K15, K34, and K55 and deleted the last six amino acids of TM to prevent intracellular retention and enhance its expression at the cell surface, as described for CI-PFV [39,48]. The original plasmid was that used to produce the foamy vector expressing GI-D468 Env [17]. Mutagenesis and cloning were performed by Genscript, The Netherlands.

### Neutralization assays

Before use in neutralization assays, plasma samples were diluted 1 to 10 in DMEM + 1 mM MgCl_2_, heated 30 min at 56°C to inactivate complement proteins and frozen as single-use aliquots. Transmitter cells (BHK-21 or HT1080) were seeded (10^5^ cells/25 cm^2^ flasks) and infected the following day at a multiplicity of infection (moi) of 0.05. Ruxolitinib (5 μM) was added to gorilla SFV -infected cultures [49]. At 72 h post-infection, SFV-infected cells were seeded in 96-well plates (5 x 10^3^/well) in triplicates. The next day, serial dilutions of plasma samples were added to transmitter cells and the plates incubated for 1 h before the addition of 5 x 10^3^ uninfected GFAB target cells. After 72 h of culture, β-galactosidase expression was detected by X-gal staining [44]. An Ultimate UV Image analyzer (CTL Europe, Bonn, Germany) was used to count X-Gal-stained cells. One infectious unit (IU) was defined as a blue cell or syncytia. Despite using the same moi on day 0 and the same transmitter-to-target cell ratio on day 4, the number of IUs was higher for chimpanzee than gorilla SFV strains and higher for HT1080 than BHK-21 cells on day 7. Data were therefore expressed as relative infectivity for easier comparison of plasma titration curves. Cells cultured without the addition of plasma samples provided the reference value, which is indicated in the figure legend. Relative infectivity was calculated for the wells treated with plasma samples and is expressed as a percentage of the reference value. Cell-free virus neutralization by the same plasma samples was carried out as described [17]. Briefly, assays were performed in triplicate in 96-well plates in which 5 x 10^3^ GFAB cells were infected with 100 IU of virus preincubated with serial dilutions of plasma samples. Neutralization titers were defined as the inverse of the plasma dilution required to reduce viral infectivity by half (IC_50_).

### Fluorescence microscopy analysis

BHK-21 or HT1080 cells were seeded (10^5^ cells/25-cm^2^ flask) and infected the following day at a moi of 0.05. At the second passage, cells were seeded on glass coverslips (borosilicate glass D263M, Marienfeld) at a density of 3 x 10^4^ cells/ml in 24-well plates. Cells were cultured until the appearance of CPE or for a maximum of three days. Cells were fixed by removing the culture medium before the addition of PBS containing 2% paraformaldehyde (PFA) and incubation for 10 min at room temperature (RT). Intercellular connections were preserved by directly adding PBS containing 16% PFA to culture medium to reach a final PFA concentration of 2%. Cells were washed with PBS and stored at +4°C in PBS.

Before intracellular staining, cells were permeabilized with 0.5% triton X-100 (Sigma-Aldrich) in PBS for 5 min, washed in PBS-0.1% Triton X-100 (PBST) and incubated with PBST-1% BSA for 20 min to block non-specific binding. SFV proteins were detected using murine monoclonal antibodies (mAbs) generated by M.L. Linial. P6G11G11 is specific for the intracytoplasmic domain of the Env leader peptide and was used conjugated to AF647 dye (anti-LP-AF647 [49], 5μg/mL). P3E10 is specific for the Env SU [50]. In ELISA assays [24], it binds to the 206-VMIDFEIPLGDPRDQ-220 peptide (OD = 1.61 vs 0.07 against the adjacent peptide for 1:100 diluted hybridoma supernatant). P3E10 was used either unconjugated (1:250 dilution of hybridoma supernatant) or conjugated to biotin (5 μg/mL). Accordingly, the secondary reagents were donkey anti murine-IgG (H+L)-AF488 (A21202, Invitrogen, 2 μg/mL) and Streptavidin-AF488 (S32354, Life Technologies, 1 μg/mL). Phalloidin-conjugated to rhodamine (R415, Lifesciences technologies, 1/250) was added to cells at the same time as the secondary reagents. We used an anti-CD98-FITC antibody (556076, BD Biosciences clone UM7F8) to stain the plasma membrane in combination with biotinylated P3E10 and Streptavidin-AF647 (S32357, Life Technologies, 1 μg/mL). The antibodies were diluted in PBST-1% BSA, the staining performed for 1 h at RT and the coverslips washed three times with PBST between each step. DAPI (0.6 μM, D9542, Sigma-Aldrich) was added followed by a 5-min incubation. Coverslips were washed with PBS, rapidly rinsed with H_2_0 and mounted on SuperFrost slides (Thermo Fisher) with one drop of SlowFade diamond antifade mountant (S36967 Invitrogen). Nail polish was used to fix the coverslips and avoid desiccation.

Before surface labelling, cells were fixed by the addition of PBS containing 16% PFA to the culture medium to reach a final PFA concentration of 2% and incubated for 1 h at RT. Staining was performed without Triton X-100 and the coverslips were incubated with the antibodies for 30 min. The SU-specific reagents were the same as for the intracellular labelling. Human plasma samples were diluted 1/100 to stain permeabilized or nonpermeabilized cells and anti-human IgG-A-M-FITC (0.2 μg/mL, 74511, Bio-Rad) was used to detect bound human antibodies.

Images were acquired using an inverted (Axio observer Z1, Zeiss) or upright (Apotome 2) widefield microscope equipped with an apotome grid and the following objectives: Plan-Neofluar 10x/0.3, 20x/0.8, 25x/0.8, 100x:1.3 Oil; Plan-Apochromat 40x/1.4 Oil and 63x/1.4 Oil. Acquired serial Z-plane frames at various Z depths with the same XY position (Z-stack) were used to build three-dimensional (3D) reconstitution images with Imaris software (Oxford instruments). Low magnification (x10) images were captured to check that the infection frequency was in the expected range (roughly 0.2 to 2%) and that cell distribution was homogenous on the 0.5 cm^2^ coverslip. For each item of interest, images from at least five different fields were acquired at high magnification (x63 or x100) for the two cell types (BHK-21 and HT1080), and three viral strains (CI-PFV, GI-D468 and GII-K74). All images were independently checked by at least two of the authors before selection of the representative ones.

For the quantification of infected cells, coverslips were stained with anti-SU-biotin, Streptavidin-AF488 and DAPI. Automatic mosaic acquisition was performed at low magnification (10x) on an area covering at least 37.5 mm^2^. All coverslips were analyzed the same day using fixed illumination parameters. The STARDIST method was used on the DAPI channel for nuclei segmentation [51] using the pre-trained versatile fluo 2D model. Maximal and minimal intensities of anti-SU staining were recorded for each segmented nucleus and then normalized (from 0 to 1) using values acquired for all samples from a given experiment. An infection status was then determined for each nucleus using the Otsu automatic threshold [52] on the normalized maximum anti-SU intensity distribution. The analysis pipeline was implemented in Python 3.7 with the scipy 3.2.4, sci-kit image 0.18.1, numpy 1.20 and pandas 1.2.4 libraries and is available at https://doi.org/10.5281/zenodo.6478121.

### Flow cytometry staining of SFV-infected cells with plasma samples

Cells (BHK-21 or HT1080) were seeded (10^5^ cells/25-cm^2^ flasks) and infected the following day at a moi of 0.05. At 72 h post-infection, cells were passed at 1:10 and 1:20 in 25-cm^2^ flasks. Starting from observation of the first syncytia in culture, infected cultures were labelled daily to assess intracellular Env expression. Cells were fixed with 2% paraformaldehyde (PFA) diluted in PBS for 10 min at RT, washed in PBS supplemented with 0.1% bovine serum albumin (BSA), permeabilized with 0.1% Triton X-100 in PBS-0.1% BSA for 10 min at RT and washed before the addition of anti-SU for 30 min at RT. Cells were washed with PBS-0.1% BSA, donkey anti murine-IgG (H+L)-AF488 was added and the cells incubated for 30 min at RT. Cells were then washed and resuspended in 300 μl PBS-2% PFA. When infection rates were above 20%, cells were used the following day for surface labelling, performed in the absence of Triton. Plasma samples (diluted 1:10) were added to cells resuspended in PBS-0.1% BSA for 30 min at RT. Cells were washed and incubated with anti-human Ig-G-BV421 (2 μg) for 30 min at RT and fixed with 300 μl PBS-2% PFA. The CI-PFV-GFP-infected BHK-21 cells were passed at 1:2 with uninfected BHK-21 and stained 2 to 4 days later using the procedures described for cells infected with primary strains. BHK-21 cells were transfected with the sENV-GFP plasmid (2.3 μg DNA per 5.10^5^ cells in the presence of LipoD293 at a 3:1 ratio) and stained two days later using the procedures described for cells infected with the primary strain.

Data were acquired using a Cytoflex cytometer (Beckman Coulter) and analyzed with Kaluza software. For the analysis, single cells were selected by gating on a FSC-A/SSC-A dot-plot. Env-specific staining was quantified by the percentage of Env-expressing cells for intracellular labelling or median fluorescence intensity (mfi) for surface labelling. Staining of SFV-infected cultures is expressed as the ratio of mfi from infected to mock-infected cultures, or ratio of mfi from GFP expressing (GFP^pos^) to GFP negative (GFP^neg^) cells to correct for nonspecific binding of plasma samples to cells.

### Live-cell imaging

CI-PFV-GFP-infected cells were mixed with uninfected cells to obtain a GFP-expressing cell frequency of 5% and seeded in 96-well plates (3000 cells/well) in triplicate. Plates were cultivated in an IncuCyte Live-Cell Analysis device and phase and green fluorescence images acquired every hour over four days with a 4x or 20x objective. Cell confluence was quantified by the Incucyte software using in-phase contrast images. We manually counted intercellular connections and measured their length using the software measurement tool.

### Statistics

We used the paired t test to compare the relative infectivity of cells and virus exposed by plasma samples. The level of significance was set to 0.05.

## Results

### Setting up of the cell-to-cell SFV transmission assay

Two cell lines, one epithelial (BHK-21) and one fibroblastic (HT1080), with the highest susceptibility to primary zoonotic gorilla SFV were used as virus-transmitter cells [49]. They were infected at a low moi for a short period of time to minimize viral particle release associated with CPE. The target cells were the indicator GFAB cell line, which produces β-galactosidase under the control of the LTR from a gorilla SFV [44]. After coculture with the transmitter cells, the infected target cells were specifically stained for the colorimetric reaction, with no signal detected in the transmitter cells. Gorilla SFV-infected transmitter BHK-21 cells were seeded at a low density to reach a final level of infection in the same range as that used in the cell-free neutralization assay [17].

**Fig 1:**
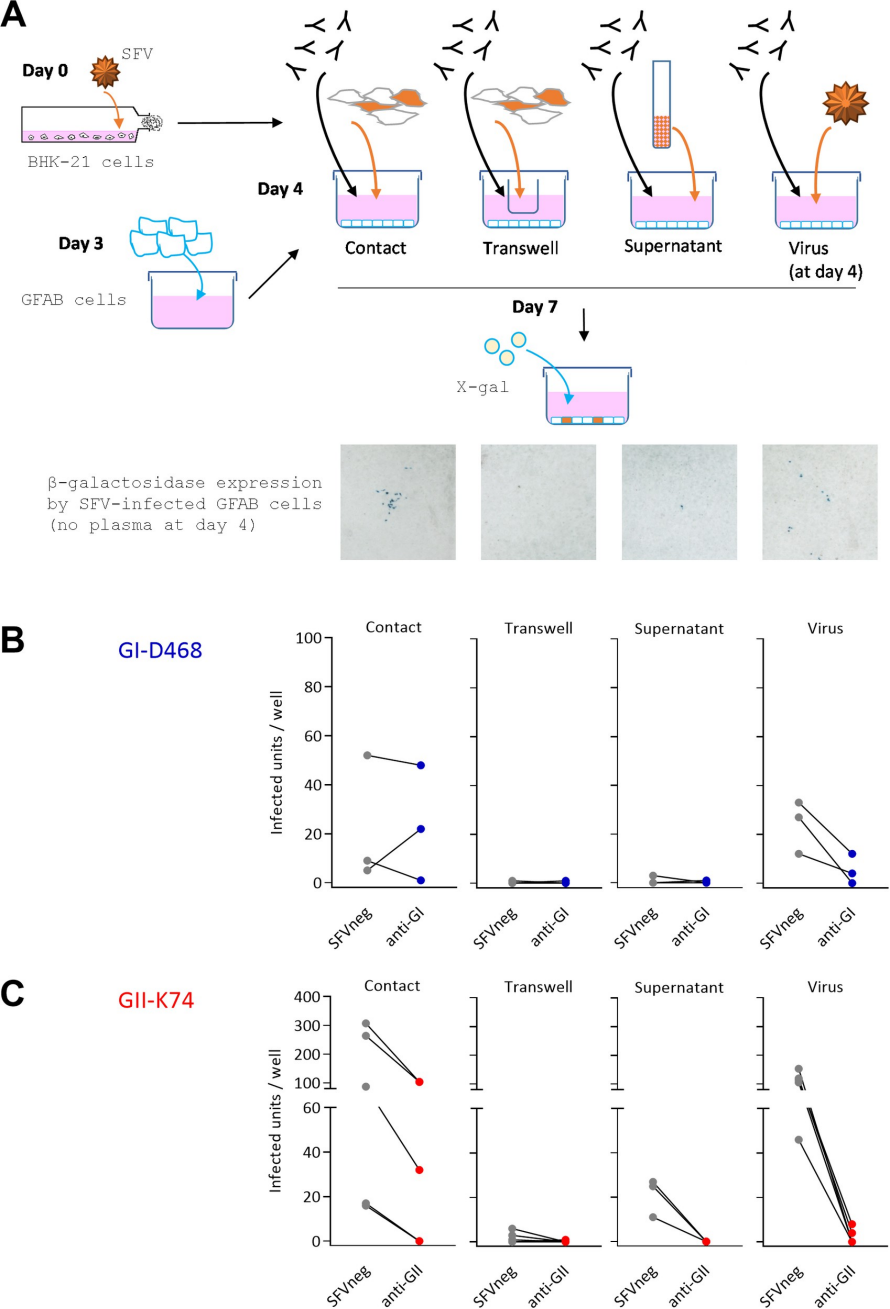
Settings of the SFV cell-to-cell transmission assay. A. Schematic description of the experiments to verify that SFV infection requires cell contact. On day 0, transmitter BHK-21 cells were infected at a moi of 0.05 for 72 h in T25 cm^2^. On day 3, uninfected indicator GFAB cells were seeded in 24-well plates (2 x 10^4^ cells/well) and infected cells were added to GFAB cells on day 4. These were placed in direct contact with the indicator cell line or in the insert of transwell devices (polycarbonate membrane-insert pore size = 0.4 μm). In addition, we quantified the infectious virus present in the supernatants of infected cells. As a control, *de novo* infection with cell-free virus was performed using the same moi as for standard neutralization assays of cell-free virus (moi of 0.02). Plasma samples were added to the culture medium at a final dilution of 1:100 on day 4, immediately after infection. On day 7, β-galactosidase expression under control of the SFV LTR was detected by addition of its chromogenic substrate (X-gal), leading to blue-stained infected GFAB cells. Representative images of wells cultivated in the absence of plasma samples illustrate the foci of infected cells after cell-cell transmission, the efficient blockade of infection by the transwell device, the low dose of infectious virus present in the supernatant of transmitter cells on day 4, and the homogenous spread of infected cells after cell-free virus infection. B. GI-D468-infected BHK-21 cells and GI-D468 virus were used to infect GFAB cells via the four transmission modes described in panel A. On day4, we added 1:100 diluted plasma from one uninfected individual (SFV^neg^, MEBAK195, gray symbols) and one neutralizing plasma (anti-GI, LOBAK2, blue symbols). The number of infectious units (i.e., cells or syncytia) per well is presented for independent experiments. C. GII-K74-infected BHK-21 cells and GII-K74 virus were used to infect GFAB cells via the four transmission modes described in panel A. On day4, we added 1:100 diluted plasma from one uninfected individual (SFV^neg^, MEBAK195, gray symbols) and neutralizing plasma (anti-GII, MEBAK88, red symbols). The number of infected cells per well is presented for independent experiments. Panel A was created with Biorender.com.

We verified that SFV infection detected in the microtitration assay required cell contact (Fig 1). No infection was detected when transmitter and target cells were separated by a membrane permeable to cell-free virus (transwell). The supernatants of transmitter cells carried little infectious virus relative to the cells themselves. Cell-to-cell and cell-free infection led to clusters of infected cells and homogenous distribution throughout the well, respectively (Fig 1A). Thus, we defined culture conditions in which GFAB cells were infected through cell-to-cell transmission, with no or minimal contribution of infection by released free viral particles. Infection with GI-D468 and GII-K74 strains was tested in the presence of a plasma sample from an uninfected individual (SFV^neg^) or neutralizing plasma samples (anti-GI and anti-GII, respectively) in the four conditions of infection: contact with infected cells, infected cells in transwells, infected cell supernatants, and cell-free virus). The neutralizing plasma samples blocked cell-free transmission and had no or a partial effect on cell-to-cell transmission (Fig 1B and 1C).

### Plasma samples from SFV-infected individuals do not neutralize SFV cell-to-cell transmission

**Fig 2:**
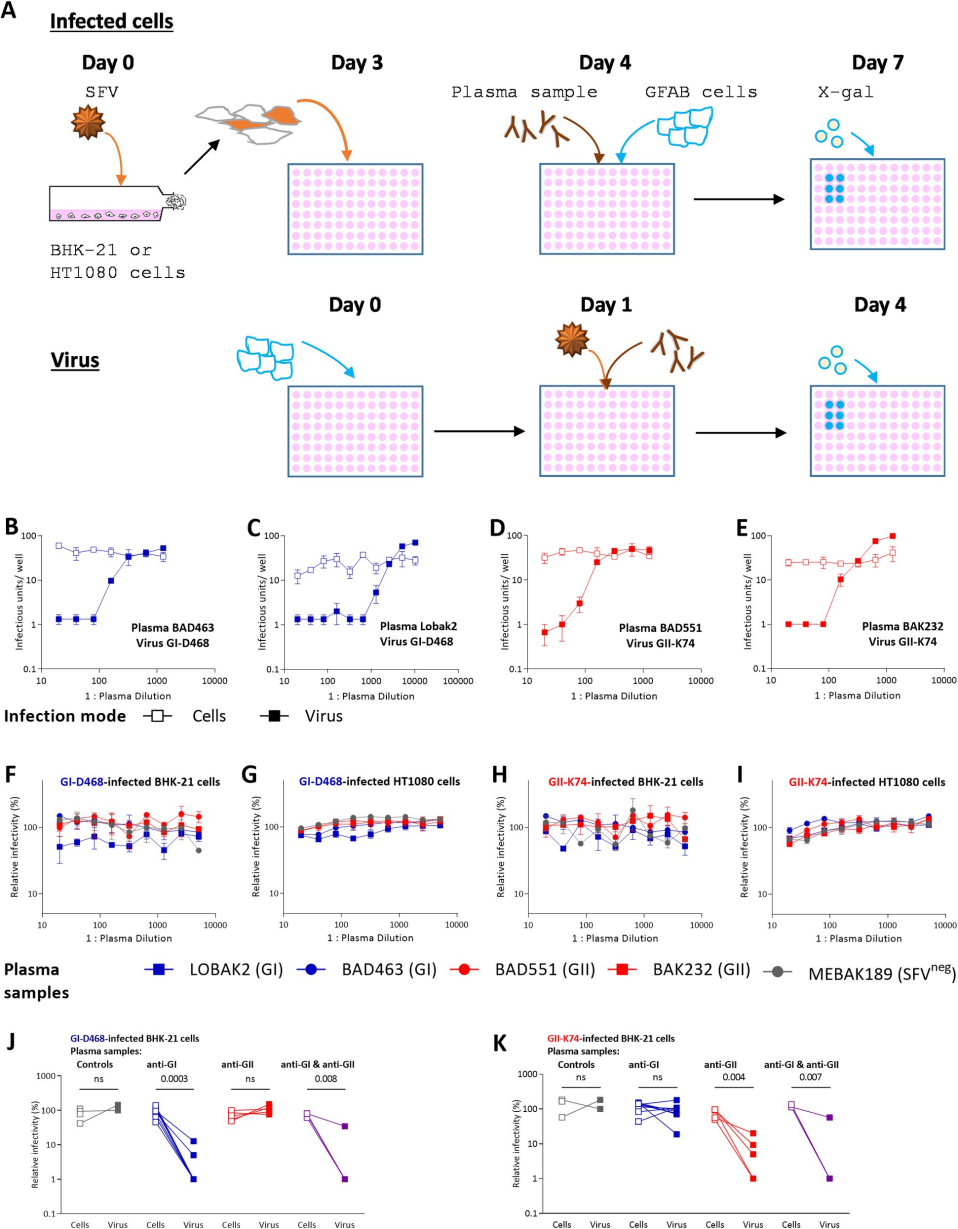
The neutralization capacity of plasma samples from SFV-infected individuals depends on the infection route. A. Schematic description of the neutralization experiments. On day 0, transmitter cells (BHK-21 or HT1080) were infected at a moi of 0.05 and seeded in 96-well microtitration plates (5 x 10^3^ cells/well). On day 1, infected cells were incubated with serial dilutions of plasma samples for 1 h before the addition of 5 x 10^3^ uninfected GFAB cells. On day 4, β-galactosidase expression by infected GFAB cells was detected by X-gal staining. For cell free virus neutralization, GFAB cells were seeded in 96-well microtitration plates at day 3. On day 4, the viral inoculum (moi of 0.02) was incubated with serial dilutions of plasma samples for one hour before addition to GFAB cells. At day 7, β-galactosidase expression by infected GFAB cells was detected by X-gal staining. B-E: Neutralization curves of BHK-21 SFV-infected cells (open symbols) and cell-free virus (filled symbols) are shown for four plasma samples against the matched genotype strain. Individuals BAD463 (B) and LOBAK2 (C) are infected with a GI strain and their plasma samples were tested against the GI-D468 strain; individuals BAD551 (D) and BAK232 (E) are infected with a GII strain and their plasma samples were tested against the GII-K74 strain. F-I: Plasma samples from BAD463, LOBAK2, BAD551 and BAK232 SFV-infected individuals and from one uninfected control (MEBAK189) were tested for the neutralization of BHK-21 (F, H) or HT1080 (G, I) cells infected with GI-D468 (F, G) or GII-K74 (H, I). In panels B-E, results are presented as IU/well to show that the infectious loads were comparable between cell-transmitted and cell-free virus conditions. IU/well values of 0 were replaced by 1 to allow visualization on the graph. In panels F to K, the results are expressed as the infectivity relative to that of cells cultivated in the absence of plasma sample (untreated cells) to show data from each virus–plasma pair on the same scale. Experiments have been carried out in triplicates and mean and standard deviation are shown. The mean number of IU/well transmitted by untreated cells were 24 (GI-D468 infected-BHK-21, panel F), 83 (GII-K74 infected-BHK-21, panel H), 978 (GI-D468 infected-HT1080, panel G) and 629 (GII-K74 infected-HT1080, panel I). In each panel, the inverse of the plasma sample dilution is presented on the x axis and the mean and standard errors from triplicates are shown. J-K. Twenty-two plasma samples diluted 1:80 were tested for their capacity to neutralize BHK-21 transmitter cells infected with GI-D468 (J) or GII-K74 (K). Results are expressed as the infectivity relative to that of cells cultivated in the absence of plasma samples. The reference values for cell-transmitted virus were 25 IU/well for GI-D468 and 83 IU/well for GII-K74 and ≈ 100 IU/well for cell-free virus according to the experimental design [17]. The relative infectivity of cell-transmitted virus in the presence of plasma samples is shown by open squares and labelled as “cells” on the x axis. Relative infectivity of cell-free virus in the presence of the same plasma sample is shown for comparison by filled squares, labelled as “virus” on the x axis. Data are presented for four plasma samples from uninfected controls (grey symbols), nine samples from SFV-infected individuals that neutralize the GI-D468 strain (blue symbols), five samples that neutralize the GII-K74 strain (red symbols) and four samples that neutralize both the GI-D468 and GII-K74 strains (purple symbols). *P* values from the paired t test are indicated in panels J and K. Panel A was created with Biorender.com.

Then, we used the microtitration assay to quantify inhibition of cell-associated and cell-free virus by serial dilution of plasma samples (Fig 2A). Two plasma samples that neutralize the cell-free GI-D468 strain (BAD463, LOBAK2) failed to neutralize GI-D468-infected BHK-21 cells (Fig 2B and 2C). Similarly, two plasma samples that neutralize cell-free GII-K74 (BAD551, BAK232) did not neutralize GII-K74-infected BHK-21 cells (Fig 2D and 2E). Importantly, without the addition of plasma samples, SFV-infected BHK-21 cells transmitted a similar or lower number of IUs than in the cell-free virus condition: GI-D468: 33 and 70 IU/well for infected cells and virus, respectively; GII-K74: 67 and 80 IU/well. Therefore, the action of neutralizing antibodies was assessed against comparable cell-free and cell-associated infectious doses.

These four samples and a fifth one drawn from an uninfected control were tested at dilutions ranging from 1:20 to 1:5,120 against two cell lines (BHK-21 or HT1080) infected with strains belonging to the two gorilla SFV genotypes (i.e., GI-D468, GII-K74). BHK-21 cells transmitted less virus than HT1080 (as indicated in the figure legend) and the results are expressed as the infectivity relative to untreated cells for easier representation. Both cell lines transmitted gorilla SFV that was resistant to neutralizing activity (Fig 2F and 2I). In addition, plasma samples failed to block infection transmitted by cells infected with the antigenically related chimpanzee SFV strains, CI-PFV and CII-SFV7 (S1A–S1D Fig).

We generalized our findings by testing additional plasma samples at a single dilution (1:80) against BHK-21 cells infected with the gorilla or chimpanzee SFV (Figs 2J, 2K, S1E and S1F). The data obtained with cell free virus are shown for comparison. None of the 22 samples neutralized the SFV infection transmitted by infected cells, despite inhibition of cell-free virus from one or both genotypes. In conclusion, plasma antibodies neutralize cell-free SFV infection but not SFV cell-to-cell transmission under closely related *in vitro* culture conditions.

### Early addition of neutralizing antibodies before *de novo* production of viral proteins reduces but does not prevent the cell-to-cell spread of SFV

**Fig 3:**
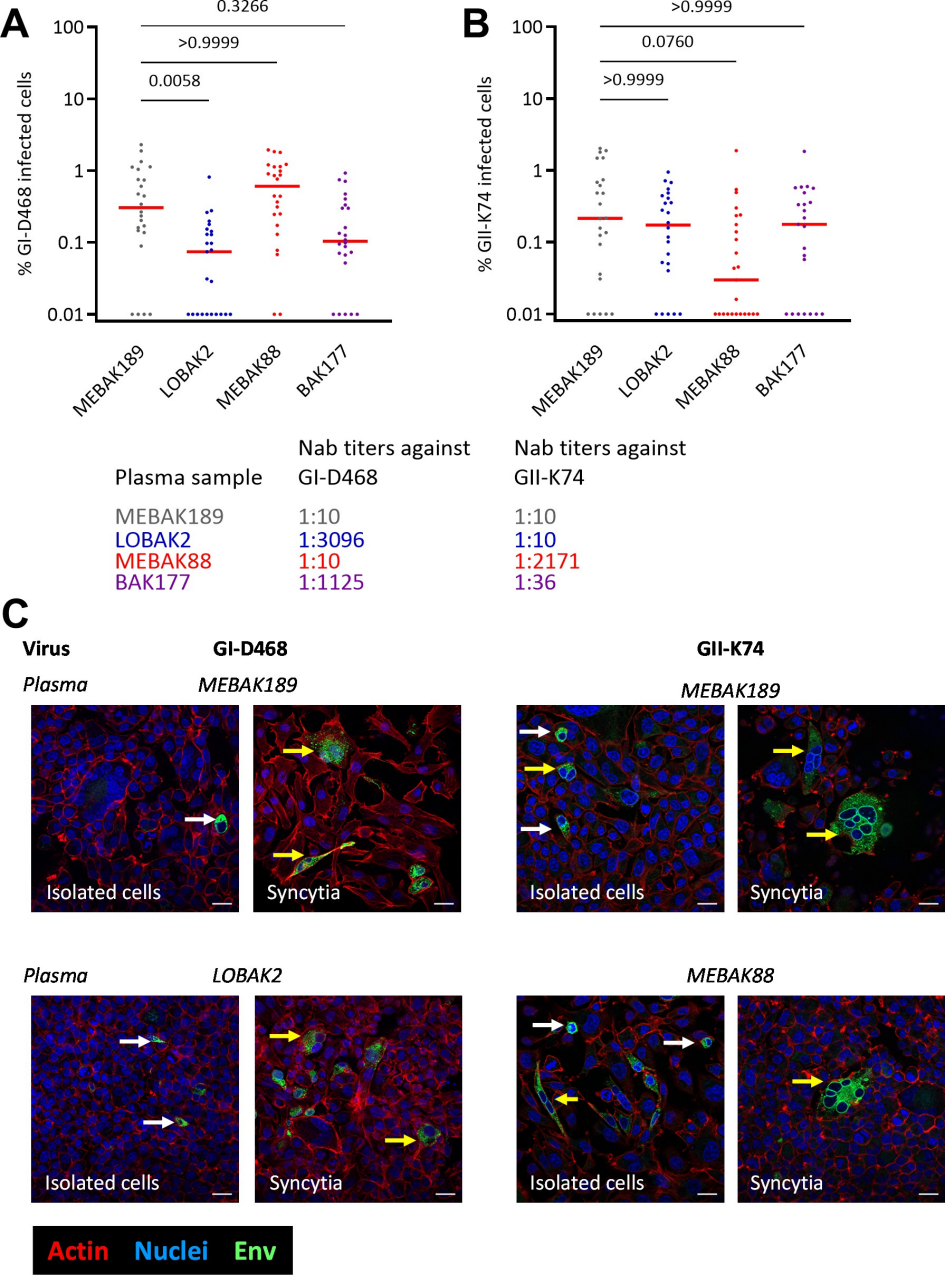
The addition of plasma samples from SFV-infected individuals before the production of viral proteins does not block cell-to-cell transmission of gorilla SFV. BHK-21 cells were infected at a moi of 0.05 with GI-D468 (A) or GII-K74 (B) for 2 h, washed and cultured in the presence of 1:100 diluted plasma samples (S2A Fig). At the last passage, cells were seeded on glass coverslips and fixed 72 h later. Cells were permeabilized with 0.5% Triton X-100 and stained with anti-SU-biotin+Streptavidin-AF488, phalloidin (actin) and DAPI (nuclei) and analyzed by brightfield microscopy at a magnification of 10x. Images covering a 37.5-mm^2^ square in the center of the coverslip were acquired. Nuclei and infected cells were quantified as described in Materials and Methods from 25 images per coverslip, corresponding to 48,000 to 140,000 analyzed cells/condition. Data are presented as the infection rate per image to reflect the variability of the infection rate across the coverslip. Plasma samples were derived from an uninfected individual (MEBAK189, grey symbols) and from SFV-infected individuals for whom the plasma neutralized GI-D468 (LOBAK2, blue symbols), GII-K74 (MEBAK88, red symbols), or both strains (BAK177, purple symbols). Their neutralization titers (IC_50_) are indicated on the figure. The Mann-Whitney test was used to compare cultures treated with samples from uninfected and SFV-infected individuals and the *P* values are indicated above the graphs. C. Representative images captured at magnification of 63x showing that the addition of non-neutralizing (MEBAK189, upper line) or neutralizing plasma samples (LOBAK2 and MEBAK88, bottom line) had no effect on the morphology of the BHK-21-infected cells, which consisted of either isolated elongated cells (white arrows) or multicellular infected foci (yellow arrows). Env, actin and nuclei are presented in green, red and blue, respectively; scale bar = 20 μm.

In the microtitration assay, infected transmitter cells were cultured for three days before incubation with plasma samples and coculture with uninfected target cells. Such kinetics may allow Env expression to reach levels at which anti-Env antibodies present in the samples are all bound to Env, leaving a sufficient amount of free Env protein to mediate viral entry. We thus tested another experimental setting (S2A Fig) in which we added plasma antibodies (SFV^neg^, anti-GI, anti-GII and anti-(GI+GII)) on BHK-21 cells two hours after exposure to cell-free virus. We detected SFV infection by immunofluorescence. Cells were labelled with anti-SU and DAPI and 45,000 to 140,000 cells per condition were analyzed (S3 Fig). Plasma samples with high neutralizing titers against the genotype-matched strain reduced the rate of infected cells by 47% (GI-D468 + anti-GI plasma), 13% (GI-D468+antiGI+GII plasma) and 70% (GII-K74 + anti-GII plasma) (Fig 3A and 3B). These reductions were inferior to those observed in the cell-free neutralization assay (> 99% at 1:100). Furthermore, the presence of plasma induced no obvious change in the size or morphology of mono- or multicellular infected foci (Fig 3C). In conclusion, neutralizing plasma antibodies added early after exposure to the virus and before *de novo* viral protein production had a very modest effect on cell-to-cell spreading of SFV.

### Cellular localization of gorilla SFV Env

**Fig 4:**
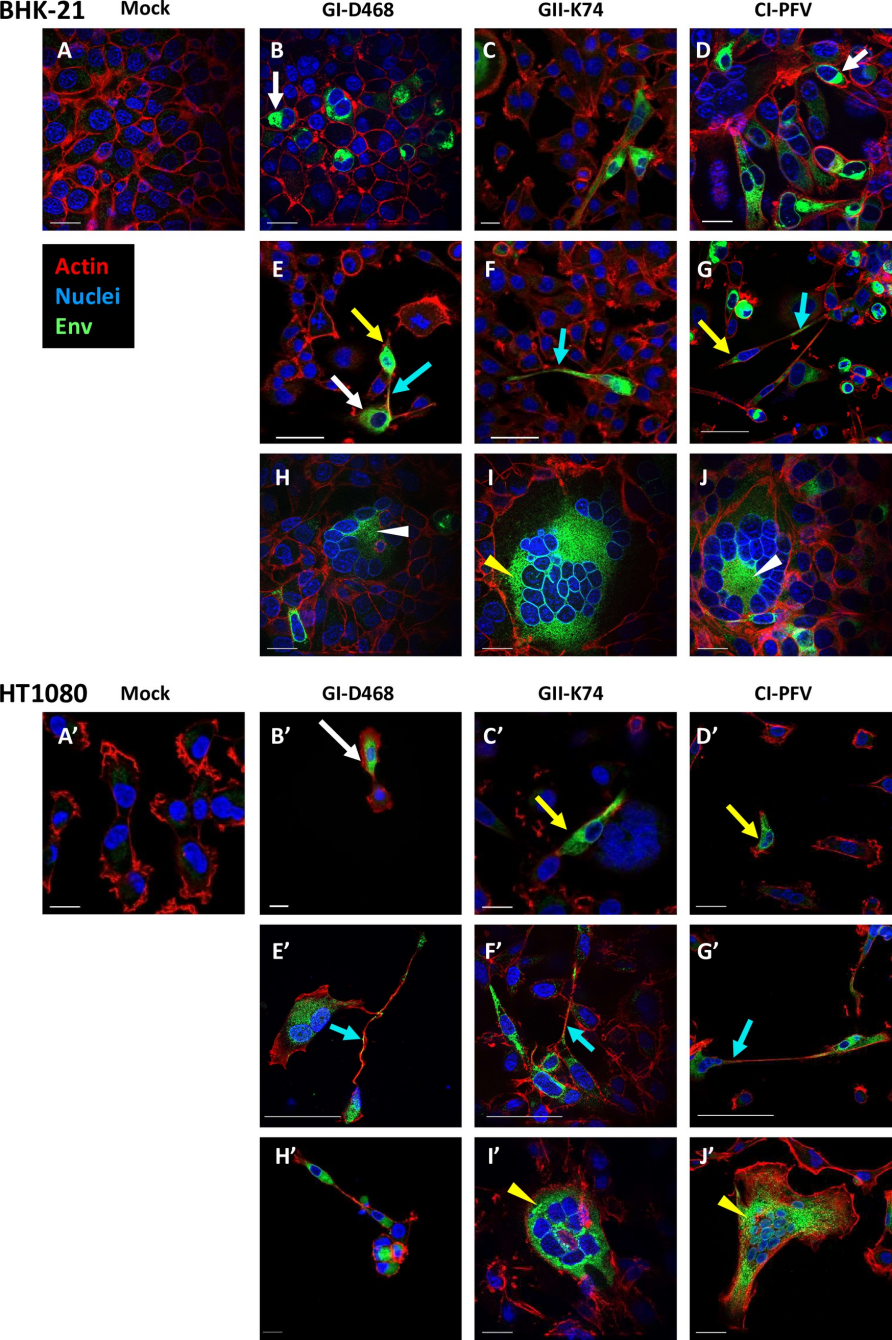
Env localization in gorilla SFV-infected cells. Cells were infected with GI-D468, GII-K74, or CI-PFV, at a moi of 0.05. At the second passage, cells were seeded on glass coverslips and fixed within 1 to 3 days, depending on the cytopathic effect (S2B Fig). Cells were permeabilized with 0.5% Triton X-100 and stained with anti-SU-biotin+Streptavidin-AF488, phalloidin (actin) and DAPI (nuclei) and analyzed by brightfield microscopy with optical sectioning. Top panels: BHK-21 cells; bottom panels: HT1080 cells (indicated as A’ to J’ for conditions matching A to J). A: Mock infected cells, B-E-H: GI-D468 infected cells, C-F-I: GII-K74 infected cells, D-G-J: CI-PFV infected cells. B-C-D: SFV Env in isolated cells is located in the cytoplasm and surrounding the nuclei and some infected cells acquired fusiform shapes. E-F-G: Intercellular connections are visible between two infected cells or between infected and uninfected cells. Env labelling is present in the intercellular connections; H-I-J: Syncytia with diffuse Env staining within a crown formed by nuclei or in the cytoplasmic space around the nuclei; both patterns were observed for the three strains and both cell lines. Representative examples of Env staining are indicated with white arrows for cytoplasmic labelling, yellow arrows for fusiform shapes, blue arrows for intercellular connections, white triangles for cytoplasmic labelling inside a crown of nuclei in syncytia and yellow triangles for cytoplasmic labelling around the nuclei in syncytia. Env, actin and nuclei are presented in green, red and blue, respectively; scale bar = 20 μm.

Env protein mediates the fusion of cellular membranes, allowing cell-to-cell transmission of SFV [39]. We hypothesized that neutralizing antibodies failed to inhibit SFV cell-to-cell transmission because of reduced availability of Env at the cell surface. FV buds both intracellularly and at the plasma membrane and viral strains differ in their capacity to bud at the cell surface [25,36–38,53]. Gorilla SFV has not yet been characterized in this respect. We, therefore, performed fluorescence microscopy analysis to visualize the cellular distribution of Env in BHK-21 and HT1080 cells infected with GI-D468 and GII-K74 (S2B Fig). We used the CI-PFV strain at the same moi for comparison. Cells were permeabilized before Env, actin and nuclei staining. The BHK-21 cells grew at a higher density than HT1080 cells (Fig 4A and 4A’) and were less susceptible to CPE induced by SFV (Fig 4B–4J and 4B’–4J’). Env was present surrounding the nuclei and in the cytoplasm in isolated SFV-infected cells (Fig 4B, 4E and 4B’, white arrows). Some SFV-infected cells adopted a fusiform shape (Fig 4E, 4C’ and 4D’, yellow arrows) and others showed intercellular connections containing Env (Fig 4E–4G, and 4E’–4G’, blue arrows). Both GI-D468 and GII-K74 induced syncytia of varying size (Fig 4H–4J and 4H’–4J’). Among BHK-21 cells, the largest contained more than 20 nuclei. In general, the observed syncytia were smaller in HT1080 cells, probably due to a greater CPE. Diffuse cytoplasmic Env staining was found within a crown formed by nuclei or surrounding the nuclei (Fig 4H–4J and 4I’–4J’, white and yellow triangles, respectively). The Env distribution, morphological changes and formation of multinucleated cells were similar in cultures infected with gorilla SFV strains and CI-PFV (Fig 4).

**Fig 5:**
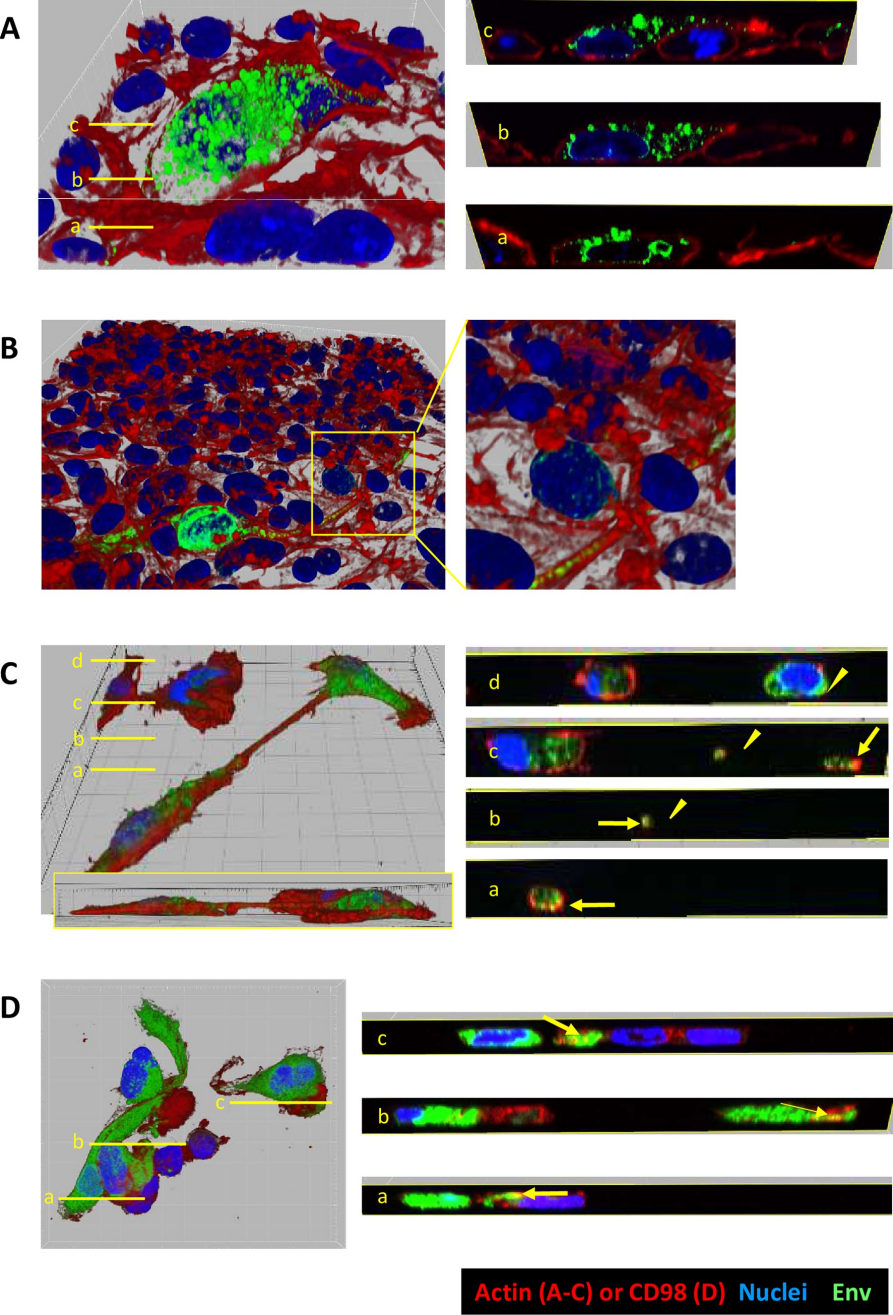
Env localization in gorilla SFV-infected cells. The infection and labeling conditions are those described in Fig 4. Acquired serial Z-plane frames at various Z depths with the same XY position were used to build 3D views with nuclei (blue), Env (green) and actin or CD98 (red) shown in a blend-rendering mode. A. GII-K74-infected BHK-21 cells, with Env staining distributed throughout multiple vesicles and around the nuclei (3D view from the top). Yellow bars correspond to orthogonal slices showing Env staining distributed throughout multiple vesicles and around the nuclei. Grid gaps correspond to 10 μm. B. Dense network of GII-K74-infected BHK-21 cells, in which an infected cell shows an elongated intercellular connection on its right side (3D view from the side). Env staining is evenly distributed despite contacts with multiple adjacent cells. Patchy perinuclear staining is observed in one cell contacting the intercellular connection (yellow square). C. Two GI-D468-infected HT1080 cells with an intercellular connection that does not adhere to the coverslip (3D view from the top side and from the side (insert)). Yellow bars indicate the position of orthogonal slices: a. cytoplasm of a fusiform cell, b-c. Intercellular connection with Env staining (triangle), d. cytoplasm and nucleus from the second infected cell (triangle). Colocalization of Env and actin is indicated by the yellow arrows. D. GI-D468-infected HT1080 cells were permeabilized with 0.5% Triton X-100 and stained with anti-SU-biotin + Streptavidin-AF647, anti-CD98-FITC, and DAPI (nuclei) (note that the colors in the figure were matched with those of the previous panels and not the conjugated fluorochromes). Yellow bars indicate the position of orthogonal slices. Colocalization of Env and CD98 is indicated by the yellow arrows.

Three-dimensional reconstitution images showed the distribution of Env in cytoplasmic vesicles and the perinuclear region (Fig 5A), the absence of polarized SFV Env staining at the contact zones between infected and uninfected cells (Fig 5B) and the formation of long intercellular connections that did not adhere to the coverslip and formed continuous cytoplasm (Fig 5C). Some Env was localized close to the actin network beyond the cell surface (yellow arrows), suggesting that Env may be expressed at the cell surface (Fig 5A and 5C). We confirmed this hypothesis by co-staining the SFV-infected HT1080 cells with anti-SU and anti-CD98 (large neutral amino acid transporter (LAT1), commonly used as plasma membrane markers) and observed colocalization of the two molecules (Fig 5D, yellow arrows).

Overall, our results show that upon infection with gorilla SFV, Env is predominantly detected in the cytoplasm of infected cells. Polarized Env localization at sites of cell contact that resemble virological synapses [54] was not observed, whereas intercellular connections containing SFV Env were frequent. Microscopy staining indicates that Env is expressed at the cell surface and the presence of syncytia indicate that their quantity at the plasma membrane is sufficient to induce cell fusion.

### Staining of SFV-infected cells by plasma antibodies reveals additional virus-induced changes in cell morphology

**Fig 6:**
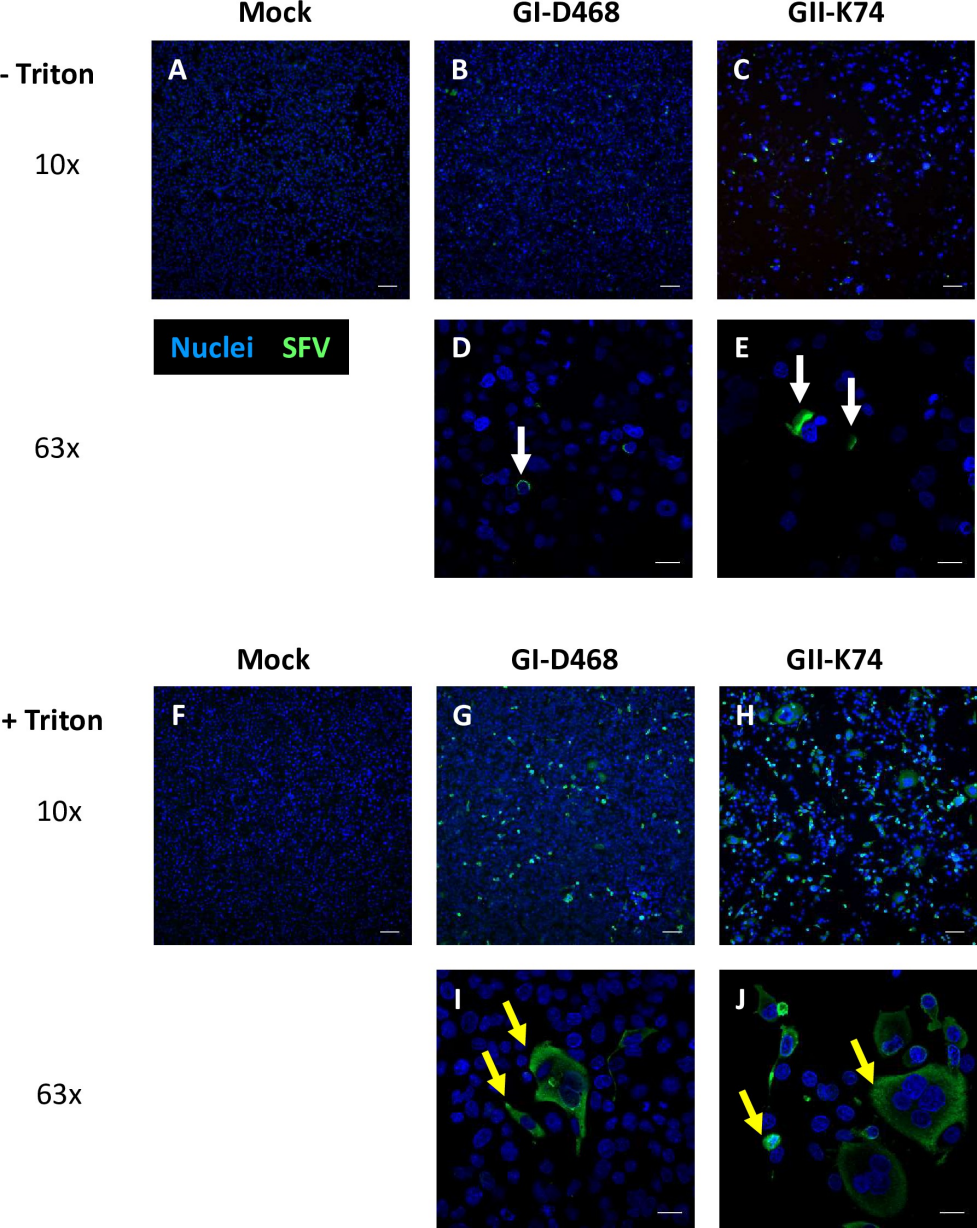
Plasma samples specifically stain SFV-infected cells. BHK-21 cells were infected with GI-D468 or GII-K74, at a moi of 0.05 for seven days, seeded onto glass coverslips and fixed within 1 to 3 days, according to the CPE (S2B Fig). Cells were either untreated (A-E) or permeabilized with 0.5% Triton X-100 (F-J) before staining with anti-GI plasma sample (LOBAK 2) diluted 1:100 and anti-human IgG-A-M-FITC and DAPI (nuclei). Cells were analyzed by brightfield microscopy with optical sectioning. A, F: Mock infected cells, B, D, G, I: GI-D468 infected cells, C, E, H, J: GII-K74 infected cells. Nuclei (blue), anti-SFV plasma (green), scale bars = 100 μm (10x magnification images) and 20 μm (63x magnification images). White arrows indicated dense SFV-specific staining localized either on the side or at the cell apex; yellow arrows indicate cytoplasmic staining.

**Fig 7:**
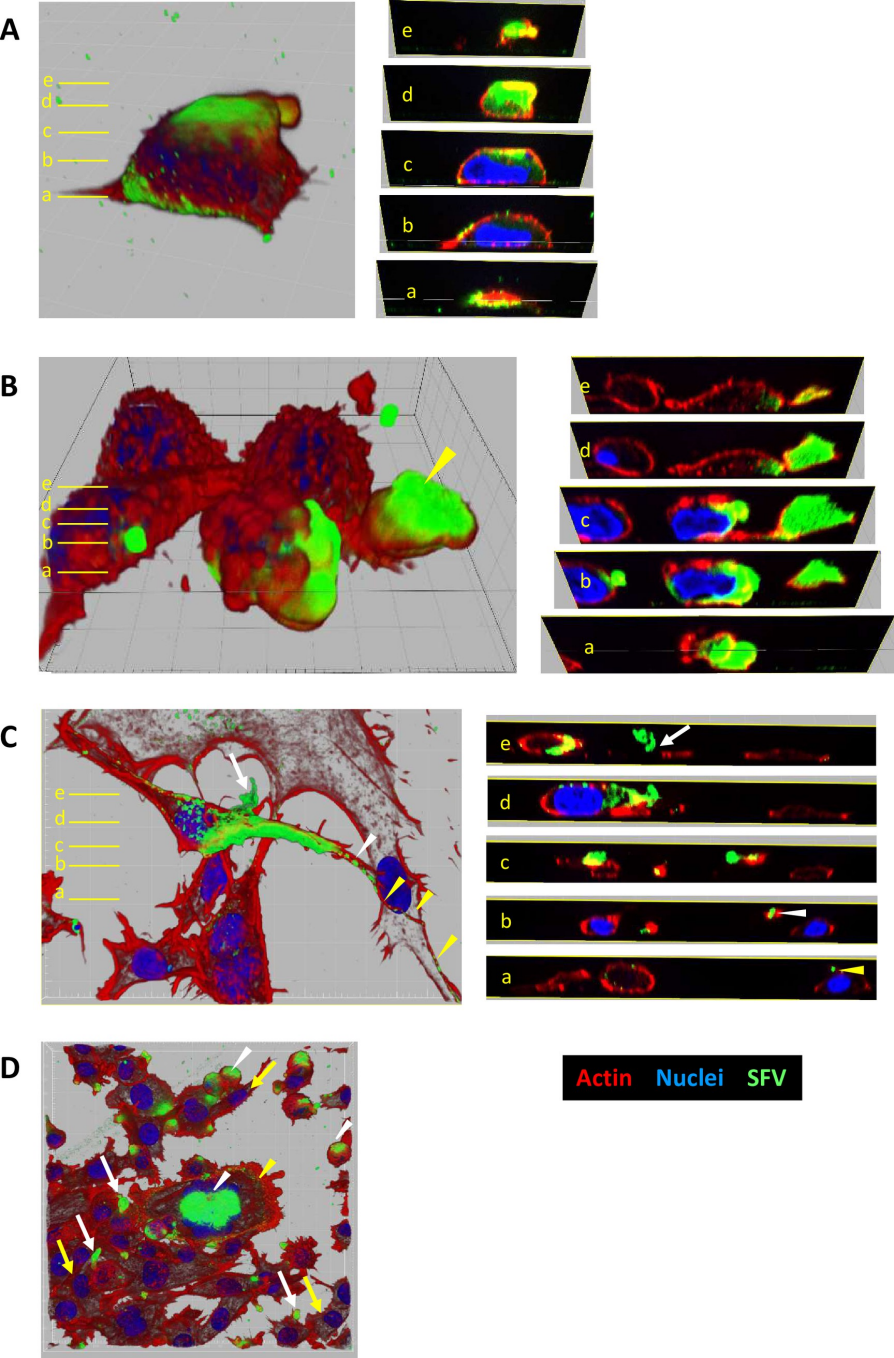
Plasma antibodies stain large surfaces, long intercellular connections and cell protrusions on SFV-infected cells. Cells were infected with GI-D468 or GII-K74 at a moi of 0.05. At the second passage, cells were seeded on glass coverslips and fixed within 1 to 3 days, according to the CPE (S2B Fig). Cells stained with anti-GI plasma sample (LOBAK2) and anti-human IgG-A-M-FITC, phalloidin (actin) and DAPI (nuclei) without incubation with Triton X-100. Labelled cells were analyzed by brightfield microscopy with optical sectioning. Acquired serial Z-plane frames at various Z depths with the same XY position were used to build 3D views with nuclei (blue), Env (green) and actin (red) shown in a blend-rendering mode. Env and actin costaining is indicated by yellow color. A. GII-K74-infected BHK-21 cell, 3D view from the side. The infected cell displays patchy SFV staining at its base, dense staining at the top and a small protrusion at the back. Yellow bars indicate the position of orthogonal slices. B. GII-K74-infected BHK-21 cells, 3D view from the top. The infected cell in the center of the image has a large cytoplasmic protrusion on its right side (yellow triangle). Yellow bars indicate the position of orthogonal slices. The protrusion has no nuclei (slices b-e), is connected to the adjacent cell (slides c and d) and does not adhere to the glass slide (slices b-e). C. GI-D468-infected HT1080 cells, 3D view from the top. An infected cell displays strong SFV staining at its surface, polarized in the direction of the intercellular connection and away from the nuclei. One densely stained protrusion is located at the center of the cell (white arrow). The long intercellular connection adheres to and wraps around the neighboring cell (yellow triangles), with extracellular SFV staining (white triangles). D. GII-K74-infected BHK-21 cells, 3D view from the top. Punctate SFV staining is observed at the basis of a syncytia (yellow triangle) and dense SFV staining at the top of cells (white triangles). Densely stained SFV protrusions (blue arrows) and several unstained cells adjacent to cells strongly expressing SFV (white arrows) are highlighted.

We analyzed gorilla SFV-infected BHK-21 and HT1080 cells labelled with plasma samples without permeabilization by fluorescent microscopy analysis. We observed dense staining localized either on the side of or at the cell apex (Fig 6A–6E). As a positive control, we permeabilized cells and observed a higher frequency of cells with cytoplasmic staining (Fig 6F–6J). Surface labelling by plasma samples was visualized in orthogonal views (Fig 7). Some intracellular SFV and actin labelling was observed, likely reflecting partial membrane permeabilization induced either by paraformaldehyde fixation, desiccation, or virus-induced membrane remodeling. The plasma samples offered the possibility to complete the studies performed with the anti-SU mAb (Figs 3 and 4), which led to a signal that was insufficient to detect Env at the surface of infected cells (S4 Fig). Anti-SFV staining was generally patchy at the base of the cell and continuous at the cell apex (Fig 7A). Cytoplasm protruded from the apex or the side of infected cells, forming anuclear compartments with dense anti-SFV staining (Fig 7B). Anti-SFV labelling was more intense on the side of cells from which intercellular connections started, as well as at the connection itself (Fig 7C). Finally, SFV-infected cells displayed dense surface anti-SFV staining without polarization at the contact zone with adjacent cells, despite the concomitant presence of syncytia in the culture (Fig 7D). Overall, SFV infection induced extensive morphological changes of cells and SFV proteins were expressed at the cell surface.

### Plasma antibodies from SFV-infected individuals recognize SFV proteins expressed at the surface of infected cells

**Fig 8:**
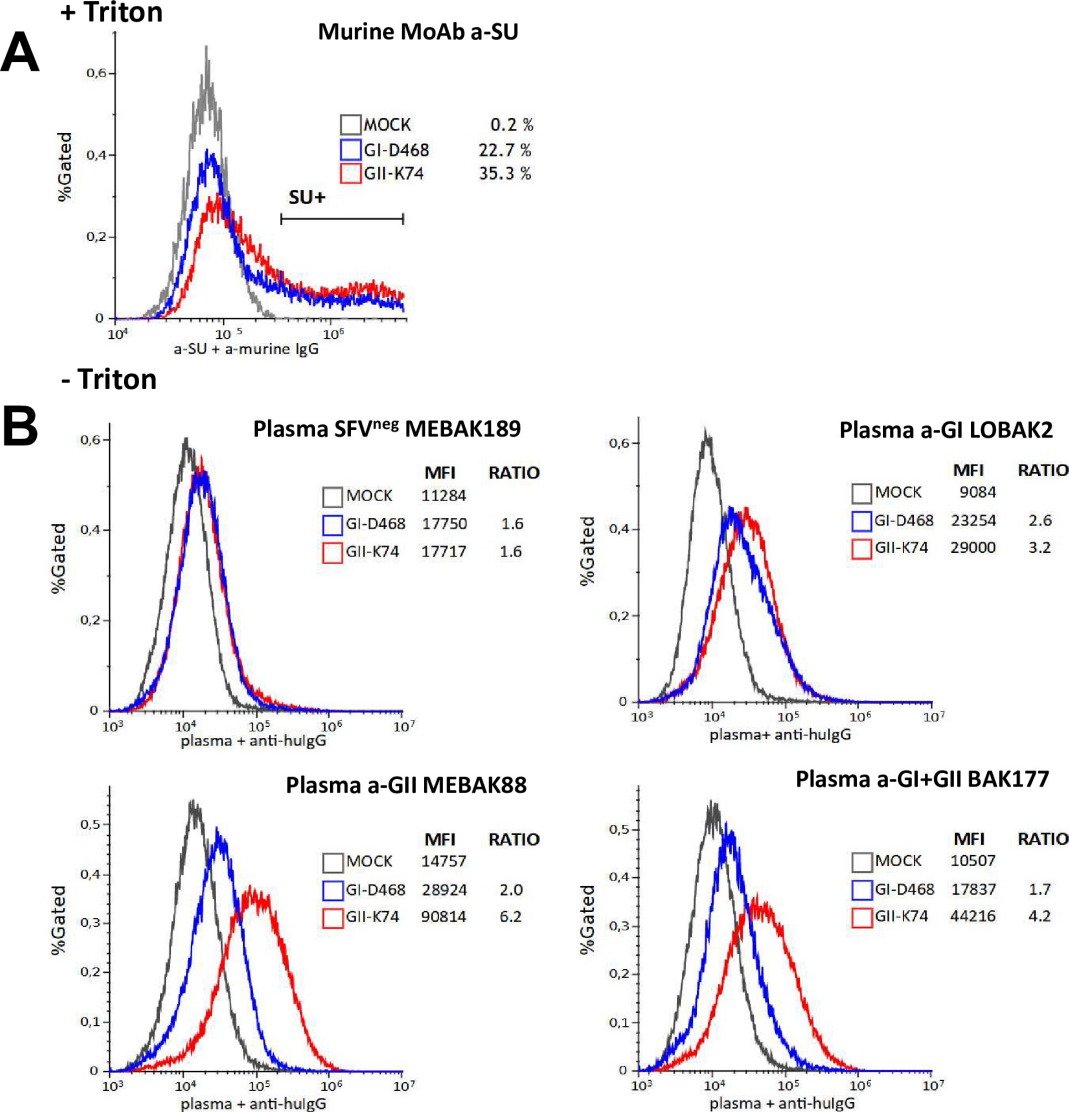
Plasma samples bind to the surface of SFV-infected cells. BHK-21 cells were infected with GI-D468 or GII-K74 at a moi of 0.05, passed twice and stained when a CPE was visible (S2C Fig). A. Cells were permeabilized with 0.1% Triton X-100 and stained with anti-SU. Env-labelled and unlabeled cells appeared as distinct peaks and Env-labelled cells were quantified as their percentage among all cells. B. Nonpermeabilized cells were incubated with four 1:10 diluted plasma samples and anti-hIgG-BV421. Staining obtained with SFV^neg^ (MEBAK189), anti-GI (LOBAK2), anti-GII (MEBAK88) and anti-(GI+GII) (BAK177) plasma samples are shown on the histogram overlay: mfi is presented on the x-axis and frequency is expressed as the percentage of gated events on the y-axis. Extracellular staining with plasma samples corresponds to an increase in fluorescence intensity of the whole cell population. Therefore, SFV-specific staining was quantified by the ratio of mfi from infected to uninfected cultures.

Then, we quantified the plasma antibodies bound to the surface of nonpermeabilized infected cells by labeling and fixing before flow cytometry analysis (Figs 8 and S2C). A global shift of fluorescence was observed in infected versus uninfected cultures. Therefore, the results are expressed as the ratio of mfi of infected versus mock-infected cultures. Immune plasma samples had ratios ranging from 1.7 to 6.2. Ratios were higher against GII-K74 than GI-D468, probably reflecting the higher infection rate. Modest background staining was observed with the plasma from an uninfected individual (ratio of 1.6). We confirmed nonspecific binding of plasma samples on uninfected cells (S5 Fig). However, samples from SFV-infected individuals showed higher staining intensity on infected cultures than on mock-infected cell cultures (Fig 8) and specific binding was dependent upon the moi of infection (as tested with CI-PFV, S5 Fig).

**Fig 9:**
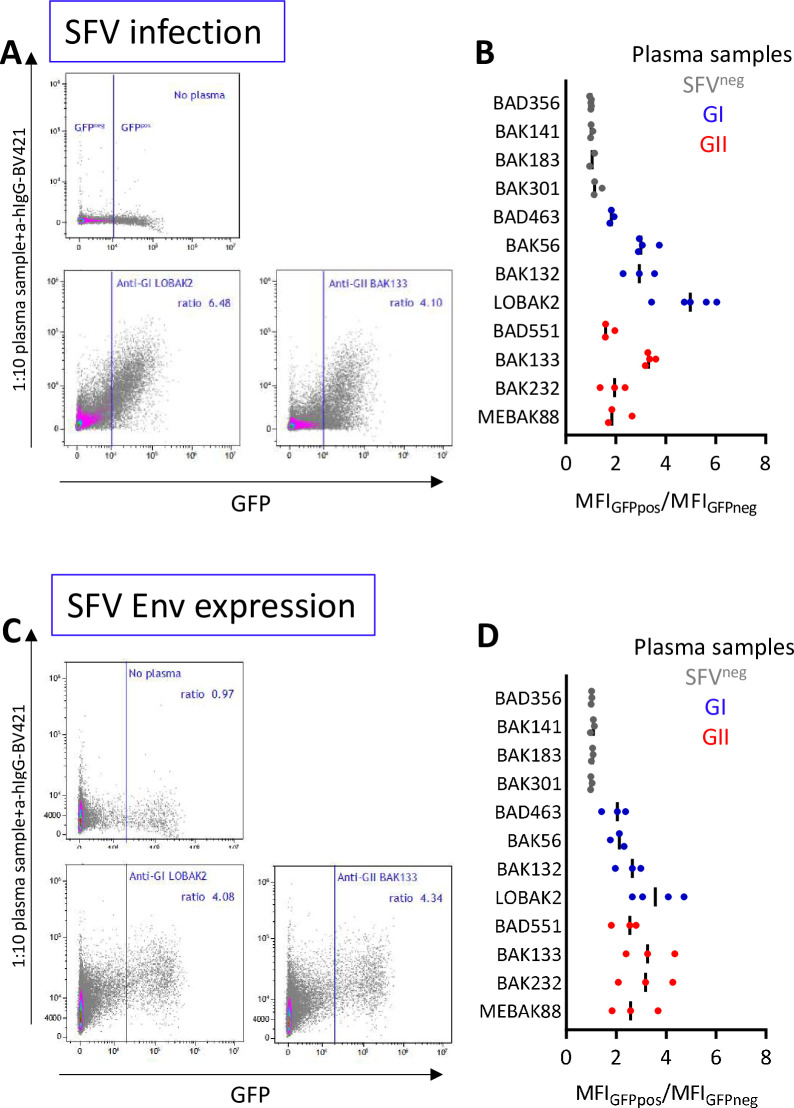
Plasma samples bind to Env expressed at the surface of infected cells. A. CI-PFV-GFP-infected BHK-21 cells were stained with 1:10 diluted plasma samples from SFV-infected individuals and anti-hIgG-BV421. Staining with human plasma samples was quantified by the mfi of GFP^neg^ and GFP^pos^ cells and the results are expressed as mfi_GFPpos_/mfi_GFPneg_ ratios (S2D Fig). B. mfi_GFPpos_/mfi_GFPneg_ ratios obtained at a 1:10 dilution for the plasma from 12 individuals: n = 4 SFV^neg^ (grey symbols), n = 8 infected with gorilla SFV belonging to genotype I (blue symbols) or genotype II (red symbols). Three to five experiments are presented per plasma sample; bars indicate median values. C. sENV-GFP-transduced BHK-21 cells were stained with 1:10 diluted plasma samples from SFV-infected individuals and anti-hIgG-BV421. Staining with human plasma samples was quantified by the mfi of GFP^neg^ and GFP^pos^ cells and the results are expressed as mfi_GFPpos_/mfi_GFPneg_ ratios (S2E Fig). D. mfi_GFPpos_/mfi_GFPneg_ ratios obtained at a 1:10 dilution for the plasma from 12 individuals: n = 4 SFV^neg^ (grey symbols), n = 8 infected with gorilla SFV belonging to genotype I (blue symbols) or genotype II (red symbols). Three to four experiments are presented per plasma sample; bars indicate median values.

We used a GFP-expressing CI-PFV molecular clone (S6 Fig) to distinguish infected from uninfected cells present in the same culture. Plasma samples were added to nonpermeabilized cells and SFV-specific binding was quantified by the ratio of the mfi of infected (GFP^pos^) versus uninfected (GFP^neg^) cells (S4D Fig). Plasma from SFV-infected individuals stained GFP-expressing cells, with stronger staining of cells expressing the highest level of GFP (Fig 9A). Twelve plasma samples were tested in at least three independent experiments. Only those from SFV-infected individuals bound to GFP^pos^ cells. The median mfi_GFPpos_/mfi_GFPneg_ ratio ranged from 1.8 to 5.0 for anti-GI samples, 1.6 to 3.3 for anti-GII samples and 1 to 1.2 for uninfected samples (Fig 9B), supporting binding of the antibodies from plasma samples to SFV proteins on the cell surface.

Finally, we transduced BHK-21 cells with a plasmid encoding GI-D468 Env fused to GFP. We introduced mutations into the LP and TM subdomains that are homologous to those that overcome CI-PFV Env intracellular retention and enhance its expression at the plasma membrane (S2E and S7 Figs [39,48]). Plasma samples from SFV-individuals stained nonpermeabilized Env-transduced cells (i.e., GFP^pos^ cells, Fig 9C). The median mfi_GFPpos_/mfi_GFPneg_ ratios ranged from 2.0 to 3.6 for anti-GI samples, 2.6 to 3.3 for anti-GII samples, and 1 to 1.1 for uninfected samples (Fig 9D). These experiments directly demonstrate the capacity of immune plasma samples to specifically bind to SFV Env expressed at the cell surface.

### Live cell imaging of CI-PFV-tagged cell-to-cell transmission

**Fig 10:**
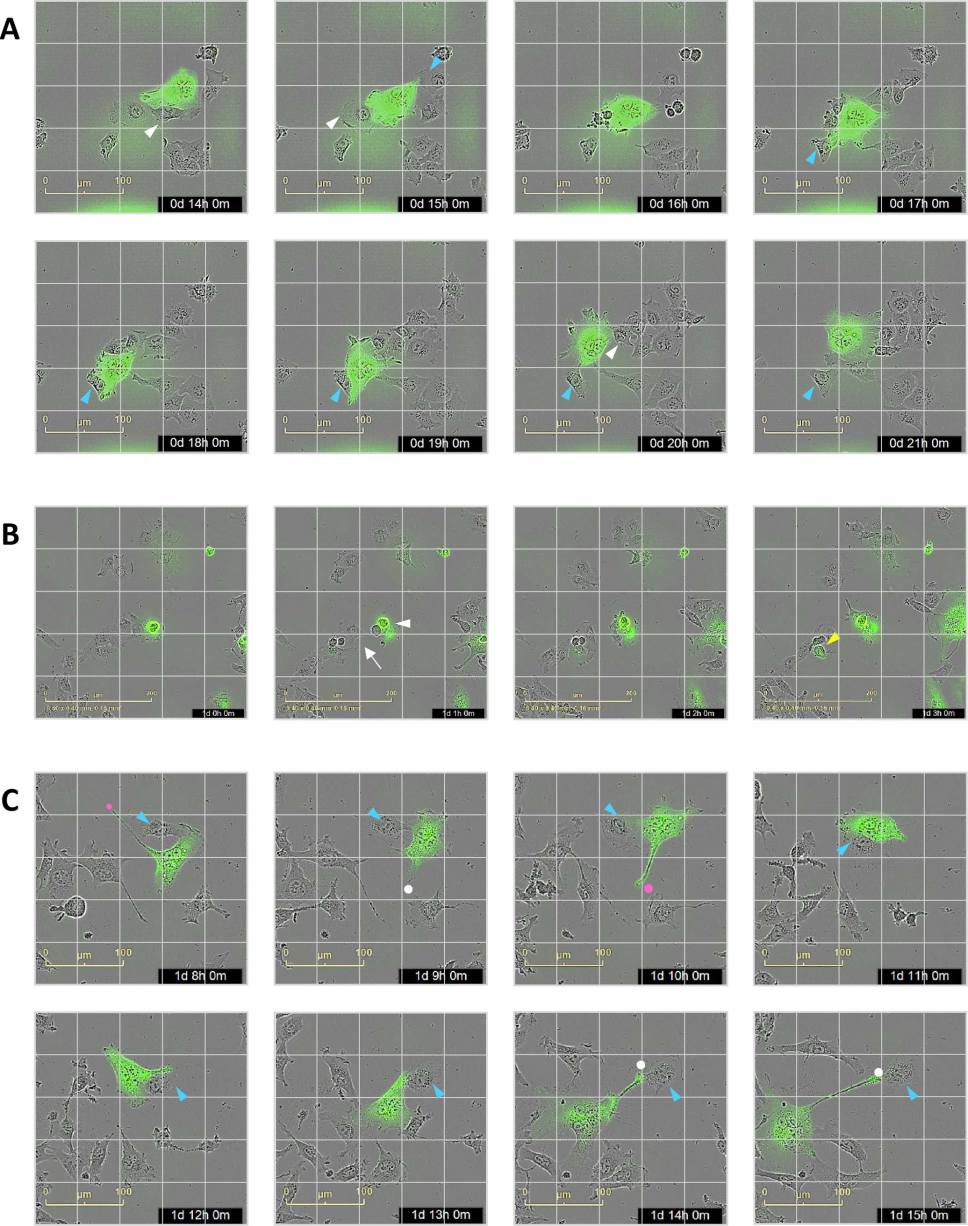
CI-PFV-GFP cell-to-cell transmission visualized by live-cell imaging. CI-PFV-GFP infected BHK-21 cells were mixed with uninfected cells to obtain a GFP^pos^ cell frequency of 5% and cultivated in 96-well plates (3000 cells/well) inside an Incucyte device for 96 h (S2F Fig). Hourly acquisition was started within 30 min after seeding. We selected sequentially acquired images (one per hour) showing representative events in CI-PFV-GFP-infected cultures. A. Acquisition between 0D-14H and 0D-20H, 250 μm x 250 μm images with a superimposed 5 x 5 grid (50 μm between lines). A GFP^bright^ syncytium has established contact with uninfected cells; its orientation frequently shifted with the lamellipodium, often oriented towards an adjacent cell. The contacts between the syncytium and cellsto fusion (white triangles indicate cells that will be fused to the syncytium in the subsequent time frame) or not (blue triangles). B. Acquisition between 1D-0H to 1D-3H, 400 μm x 400 μm images with a superimposed 5 x 5 grid (80 μm between lines). A round GFP^bright^ cell is in contact with a layer of uninfected cells at 1D-0H. One hour later, one adjacent cell expressed GFP (white triangle) and the cell cluster has split (arrow). Three hours later, among the cluster which left the GFP^pos^ cells, one cell becomes GFP^low^ (yellow triangle). C. Acquisition between 1D-8H and 1D-15H, 250 μm x 250 μm images with a superimposed 5 x 5 grid (50 μm between lines). Morphology of a migrating infected mesenchymal, with a large lamellipodium that often splits, leading to changes in direction. The uropod appears to remain anchored, either to a cell that was in contact hours before (white dot) or to the substratum (pink dot). The former interaction can generate structures that resemble intercellular connections. One cell has remained in contact with the syncytium without fusion nor productive infection (blue triangle).

Finally, we used the CI-PFV-GFP virus and live-cell imaging to obtain a dynamic view of SFV cell-to-cell transmission (S2F Fig). Contacts between infected and uninfected cells showed three major outcomes: cell fusion, transmission of productive infection to the uninfected cell without fusion, and neither fusion nor productive infection (S1 and S2 Movies). Selected sequential frames show representative fusion and non-fusion events between infected and uninfected cells (Figs 10A for BHK-21 cells and S8A for HT1080 cells; note the difference in scale and greater cell displacement in HT1080 than BHK21-cells). We readily observed SFV production after a nonfusogenic contact with an infected cell for BHK-21 cells (Fig 10B). Such an event was not observed for HT1080 cells, which show the highest susceptibility to SFV infection [30] and the highest cytopathic response (see below).

**Fig 11:**
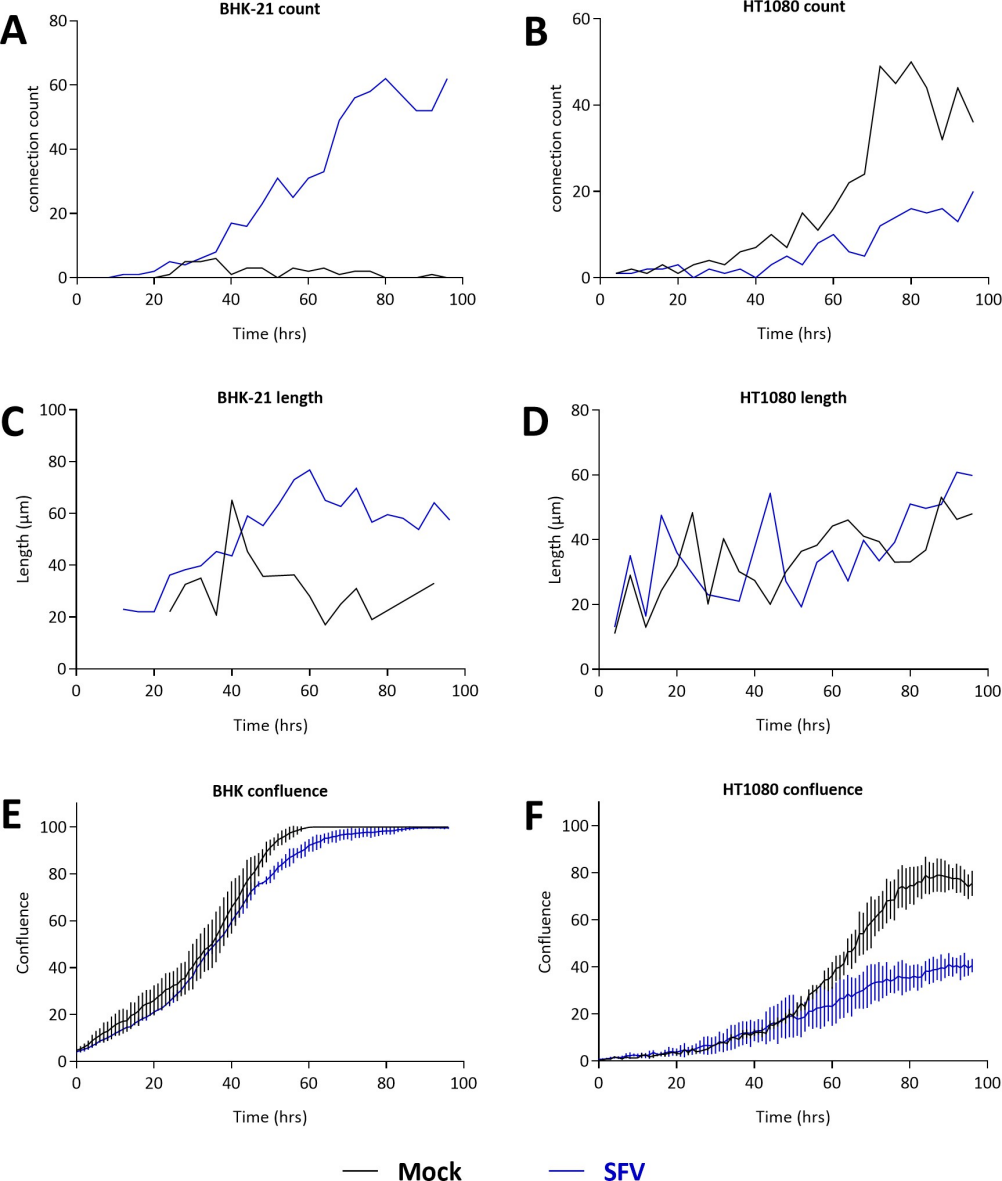
Number and length of intercellular connections in SFV-infected BHK-21 and HT1080 cells. CI-PFV-GFP-infected BHK-21 and HT1080 cells were mixed with uninfected cells to obtain a GFP^pos^ cell frequency of 5% and cultivated in 96-well plates (3000 cells/well) inside an Incucyte device for 96 h. Uninfected cells were seeded at the same density. For each condition, one 57 mm^2^ field acquired at 20x magnification, at 4-h intervals over 96 h, was randomly selected to manually count intercellular connections and measure their size. Cell confluence in the same wells was quantified by Incucyte software hourly from 0 to 96 h and expressed as the percentage of the total well surface. Intercellular connections counted per field (A-B), their mean ± SD length (μm, C-D), and cell confluence (percentage, E-F) are presented for BHK-21 (A, C, E) and HT1080 cells (B, D, F). Mock infected cultures are presented in grey and SFV infected cultures in blue.

SFV-infected cells showed higher mobility than uninfected cells, in particular at high cell density. The net displacement of BHK-21 and HT1080 cells was < 50 μm over one hour and < 100–150 μm over 12 hours at low density (S9 Fig and S3 and S4 Movies). Similarly, most isolated SFV-infected cells showed motility but little net movement (Fig 10 and S1 and S2 Movies). In subconfluent cultures, syncytia moved rapidly over a distance of > 200 μm as they fused to other cells. Intercellular connections were mostly formed after cell contact and while cells were moving apart: infected cells maintained adhesion to focal points while their bodies moved away, leading to the formation of trailing membrane extensions that persisted over 1 to 4 hours (Figs 10C and S8B, and S1 and S2 Movies). In addition, cellular extensions appeared to correspond to areas of tight attachment of the uropod to the substrate (Figs 10C and S8B, dots). When quantified at the culture level, the epithelial BHK-21 cells formed few intercellular connections and SFV infection led to an increase in their number and length (Fig 11A–11C). Cell confluence was almost similar for infected and uninfected BHK-21 cells (Fig 11E). Fibroblastic HT1080 cells spontaneously formed intercellular connections (Fig 11B) and SFV-infected HT1080 cells emitted elongated structures (S8B Fig and S2 Movie). At the culture level, the confluence of infected cells was less than that of uninfected cultures, as was the number of intercellular connections (Fig 11B and 11F). The mean length of intercellular connections appeared to be similar for uninfected and SFV-infected cell cultures (Fig 11D). These data highlight the high susceptibility of HT1080 cells to the cytopathic effect induced by SFV. Overall, live-cell imaging showed that SFV is transmitted when adjacent cells establish contact over large surfaces, whereas intercellular connections are formed posterior to cell contacts.

## Discussion

**Fig 12:**
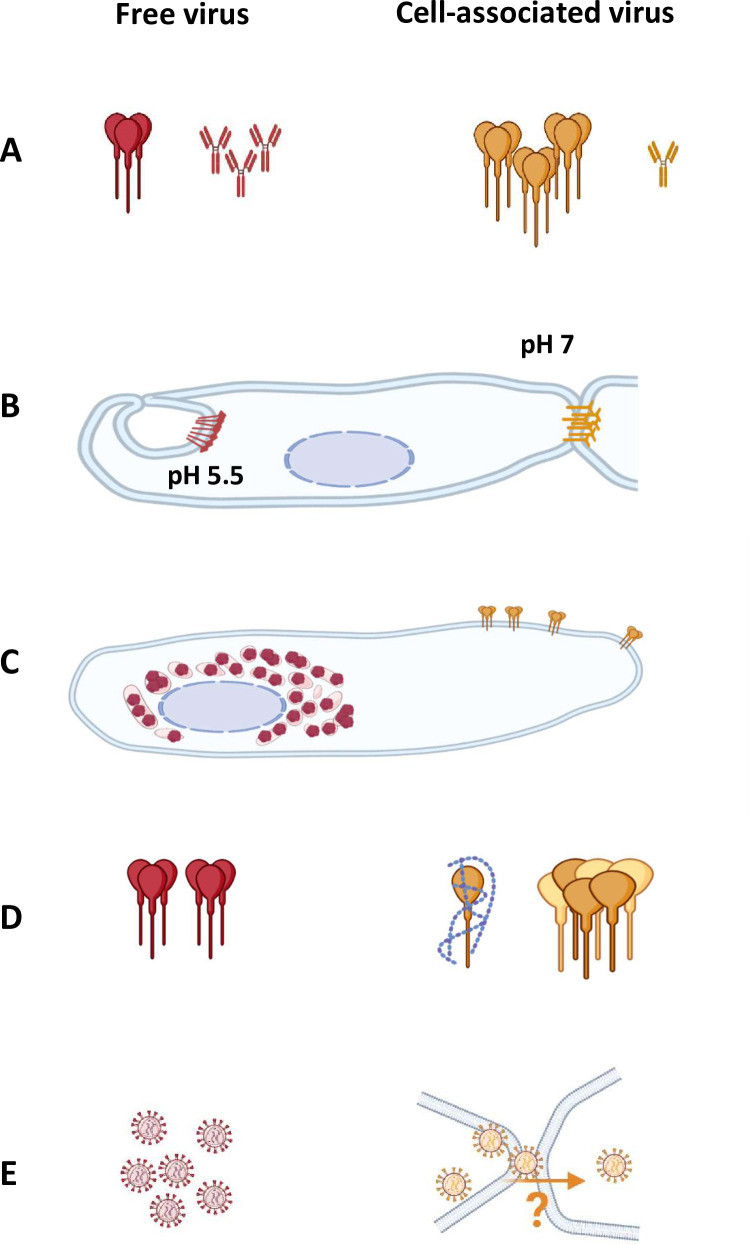
Hypotheses to explain the resistance of SFV cell-to-cell transmission to plasma antibodies. Schematic representation of the five hypotheses; cell-free virus is presented on the left side in red and cell-associated virus on the right side in orange. A. Env molecules bind all neutralizing antibodies, leaving sufficient free Env to mediate cell-to-cell fusion. Cell-to-cell virus transmission occurs at a higher multiplicity of infection than infection with cell free particles [58]. B. Viral particle entry and cell-to-cell spread rely on distinct Env properties: Env-mediated fusion depends upon an acidic pH [32] and cell fusion is restricted at physiological pH [33]. Cell-to-cell transfer of viral capsids and genomes is independent of the fusogenic activity of Env [62]. C. Env expression at the plasma membrane is restricted: Intracellular retention of Env probably acts as an immune escape mechanism because it affects the syncytium formation but not infectivity of viral particles [34,86]. D. Altered Env conformation or interference from host molecules [58,60]. E. Uncharacterized cell structures protect viruses from neutralizing antibodies. Images were created using biorender.com.

We show here that plasma antibodies from Central African hunters do not block the cell-to-cell spread of SFV *in vitro*, while still being able to neutralize cell-free virus. We also show that cells infected with gorilla SFV strains isolated from infected humans express Env at their surface and that plasma antibodies from SFV-infected individuals bind to Env at the cell surface. Expression of functional Env at the cell surface is supported by the presence of fused cells and syncytia. The mechanism of cell-to-cell spreading of gorilla SFV has not yet been defined. Here we describe novel elements: (1) contact between infected and uninfected cells may result in cell fusion, viral transmission without cell fusion, or the absence of viral transmission and/or productive infection; (2) Env accumulation at a synapse-like structure is not observable at the contact zone between cells; and (3) the frequently occurring intercellular connections mostly result from infected and/or uninfected cells moving apart after having been in contact. These features contrast with the cellular structures (synapses and nanotubes) that promote the cell-to-cell spreading of other retroviruses [55,56]. The escape of cell-to-cell spreading of SFV from neutralization could be explained by several hypotheses, summarized in Fig 12 and discussed below.

The capacity of plasma antibodies to inhibit cell-to-cell viral transmission may be affected by the high moi during cell-to-cell transmission of the virus. The neutralizing antibody to Env ratio is indeed lower in cell-to-cell than in cell-free transmission (Fig 12A [54,55,57,58]). The relative efficiency of both SFV transmission routes is unknown and we normalized the inoculum based on infectious events observed three days post-infection. In particular, we carefully attempted to avoid the saturation of antibodies by an excess of Env expressed by infected cells. In the microtitration assay, we used a moi and duration of culture at the limit of detection for the gorilla SFV strains. We first added plasma antibodies at the beginning of culture with indicator target cells, i.e., after three days of infection of the transmitter cells and found no neutralizing activity under these conditions. The addition of plasma antibodies immediately after exposure to viral particles modestly reduced the infection rate. This effect may correspond to the inhibition of cell-to-cell spreading or blockade of the entry of particles attached to the cells at the time of plasma addition. A limitation of our experimental setup is that cell-specific factors may affect Env expression at the cell surface and their susceptibility to antibodies, such as attachment/receptor molecules and restriction factors that act at the plasma membrane [59–61]. Here, we used a slowly replicating gorilla SFV in the two susceptible cell lines—BHK-21 and HT1080—that allowed their quantification over one week of infection. We indeed observed that the two cell lines differ in their ability to transmit SFV, with HT1080 being more efficient than BHK-21. Thus, we cannot exclude that some antibodies may be able to restrict cell-to-cell transmission between primary cells. To avoid non-specific inhibition and spare the rare plasma samples, we used diluted samples, matching conditions of cell-free virus neutralization and avoiding a non-specific effect of the plasma [17]. In conclusion, although we cannot formally exclude a certain level of inhibitory activity of plasma samples against cell-to-cell transmission of SFV, it is surely much less than that against viral particles.

The susceptibility of cell-free and cell-to-cell infection to neutralizing antibodies may differ because the two routes of viral spread rely on distinct Env properties. Fusion between viral and cellular membranes occurs in the endosome and is pH-dependent, except for the CI-PFV strain [32]. The conformation of Env may thus differ in the endosome and at the plasma membrane (Fig 12B). Indeed, Env-mediated fusion between adjacent cells was proposed to be restricted because it may require an acidic pH [33]. This may explain why cells in contact with infectious foci were not systematically infected (Figs 5 and 10). The interpretation of some of our observations is limited because no mechanistic data are currently available on SFV Env-mediated cell-cell fusion. Concerning the fusion of viral and cell membranes, there is still knowledge gap to be filled regarding the molecular basis that control the pH dependence of Env-mediated virus-cell fusion, which varies between FV species [33]. Second, Env-mediated cell-to-cell transfer of the genome has been demonstrated for CI-PFV. Interestingly, fusion-deficient Env, which has lost its capacity to mediate vector particle entry into susceptible cells, can support cell-to-cell transfer of the genome [62]. If such genome transfer occurs for gorilla SFV strains, it may allow resistance to neutralizing antibodies. The escape of cell-associated SFV from neutralization by antibodies may result from intracellular budding, as shown by the presence of Env in the perinuclear region and intracytoplasmic vesicles, consistent with prior knowledge (Fig 12C). The capacity of FV particles to bud at the cell membrane varies among strains and cell types. Such variation has been well described for the BFV Riems isolate. The *in vitro* selection of BFV Riems able to infect a bovine cell line through the cell-free route was associated with increased particle budding at the plasma membrane but decreased induction of syncytia [25]. Thus, Env-mediated cell-to-cell spreading and particle entry may rely on distinct mechanisms that are yet to be defined.

One alternative explanation for the lack of inhibitory action of plasma antibodies on the cell-to-cell spreading of SFV is that Env is expressed at insufficient levels or is not fully accessible to neutralizing antibodies (Fig 12D). Several viruses reduce Env expression at the plasma membrane to escape antibodies through spontaneous or antibody-mediated Env internalization [63]. Env localization is regulated by endoplasmic reticulum retention signals located in the membrane-spanning and cytoplasmic domains of LP and TM [34,39,64]. Here, we show SFV protein expression at the cell surface using plasma samples for microscopic analysis. These proteins are probably exclusively Env, based on our current knowledge of SFV infection. Cell-surface labelling was asymmetric, being polarized towards the top of cells and in the direction of intercellular extensions. In addition, we observed large protrusions. Omitting the permeabilization step before incubation with the plasma samples clearly modified the staining pattern (Fig 6) and allowed visualization of SFV proteins at the cell surface. However, concomitant limited intracellular staining occurred, despite carefully avoiding cell desiccation. Our observations call for further cellular studies to reassess intracellular and plasma membrane particle budding and their role in transmission of the virus.

Here, we demonstrated that Env from zoonotic gorilla SFV is expressed at the surface of infected cells and that plasma antibodies bind to infected cells by flow cytometry. This technique avoids background intracellular staining, as cells are kept in solution during fluorescent signal acquisition and provides a quantitative measure of antibodies bound to cells. Plasma antibody binding to infected cells occurred at levels in the range reported for a panel of broadly neutralizing monoclonal antibodies that bind to HIV-1 infected cells. Median binding levels ranged from < 2 to 4, despite HIV-1 Env budding at the plasma membrane [65]. This result argues against limited Env expression as the dominant mechanism for the resistance of SFV cell-to-cell transmission to antibodies. Clearly, certain plasma antibodies that target epitopes on LP, TM and the conserved subdomain of SU may bind to cells, as plasma antibodies from individuals infected with a genotype II gorilla SFV bind to cells infected with the genotype I chimpanzee SFV [17]. Interestingly, the only anti-SU mAb available recognized intracellular Env but not Env expressed at the surface of infected cells (S4 Fig). This antibody reacts with a peptide in ELISA and probably targets a linear epitope that is altered or masked in mature Env trimers. The recognition of cells transduced by the sENV-GFP plasmid by the anti-SU monoclonal antibody (S4 Fig) may reflect a greater quantity of Env at the cell surface due to deletion of the intracellular retention signal, or a different conformation in the absence of an interaction between intracytoplasmic LP and Gag. Comparison of the Env epitopes presented at the surface of particles and cells, particularly those located in the SUvar domain targeted by plasma neutralizing antibodies, will require monoclonal antibodies that are not yet available.

The cell-to-cell spreading of retroviruses displays varied susceptibility to neutralizing antibodies [66]. HIV-1 cell-to-cell spreading is susceptible to neutralizing antibodies with qualitative and quantitative differences relative to cell-free infection [55,58]. HTLV-1 spreads exclusively by the cell-to-cell route, which is susceptible to antibodies [67–69]. Here, we show that SFV cell-to-cell spreading is resistant to neutralization by antibodies present in human immune plasma samples that efficiently block viral particle entry. Although the ability of FV to spread by the cell-to-cell route is well established, the underlying mechanisms have not been explored, except Env-mediated cell fusion [39]. Here, we observed frequent intercellular connections between infected cells using primary gorilla SFV, as well as between infected and uninfected cells. Many viruses hijack cellular protrusions to spread between cells. These intercellular connections are heterogenous in terms of their mode of formation, width and length and the nature of the cell contact [66,70–72]. Of interest, heparan sulfates (HS) are enriched on filopodia and are required for HSV-1 surfing through the cell body, where they enter into the cytoplasm [73]. As for HSV-1, SFVs use HS as an attachment factor [30,31]. Among retroviruses, membrane protrusions of murine leukemia viruses (MLV), HTLV-1 and HIV-1 have been described: some are open ended, allowing the transfer of viral material without exposure to the extracellular surface while others are closed ended and Env-receptor interaction is required for cell-to-cell infection [74–78]. In addition, viruses can move along such protrusions while being fully exposed to neutralizing antibodies. HIV-1 Nef, and HTLV-1 p8 proteins induce cellular protrusions and promote transmission of the virus [56,76,79]. Our live-imaging data showed that among SFV-infected epithelial and fibroblastic cells, intercellular connections were generated between previously connected cells when they separate from each other, as previously described [53,54]. Therefore, the membrane extensions may not act as structures that actively promote viral spread, as described for other viruses. Rather, they might result from the increased adhesion of SFV-infected cells. Such activity has not yet been described for any SFV proteins. In conclusion, cell fusion, and possibly additional uncharacterized cellular structures, may allow cell-to-cell spreading of SFV to escape from the action of antibodies (Fig 12E).

For the purpose of assessing plasma antibody binding to infected and uninfected cells without permeabilizing them, we generated a GFP-encoding molecular clone of CI-PFV. We chose to fuse the *gfp* gene to the N-terminus of *env* because such a construct is functional in the context of foamy viral vectors [33]. This novel tool allowed us to define the infection status for each cell present in the SFV-infected cultures and obtain a more accurate assessment of plasma antibody binding than by comparing the staining of infected and uninfected cells. Another molecular chimpanzee SFV clone has recently been constructed in which the *gfp* gene was inserted in place of the *orf2* reading frame [80]. The success of both strategies to generate tagged replicative virus should be useful for further studies.

Our finding that plasma antibodies from SFV-infected individuals bind to infected cells opens new perspectives on the immune control of SFV. Cell binding by neutralizing and non-neutralizing antibodies is a prerequisite for the triggering of Fc-mediated destruction of infected cells by antibody-dependent recruitment of molecular or cellular effectors, i.e., complement, phagocytic and cytolytic cells [81,82]. One key study showed that passive transfer of plasma antibodies blocked SFV cell-associated transmission through transfusion in macaques [83]. In this *in vivo* experiment, antibodies bound to infected cells may have directly prevented SFV cell-to-cell transmission or may have recruited complement and/or cells that destroyed the infected cells. The relationship between antibody binding to an infected cell and its destruction is heterogenous and depends on the epitope targeted [58,84,85]. Two important future objectives to define the SFV-specific humoral response will thus be to (1) compare the recognition of Env epitopes on viral particles and at the surface of infected cells using monoclonal Abs and (2) assess whether plasma antibodies mediate the destruction of SFV-infected cells in the presence of molecular or cellular effectors.

## Supporting information

S1 Table Demographics and SFV infection status of individuals whose plasma samples were tested in the study. (DOCX)

S1 Fig Plasma samples from SFV-infected individuals do not inhibit cell-to-cell transmission of chimpanzee SFV strains. Experiments were performed as described in Fig 2. Transmitter cells were infected at a moi of 0.05 for 72 h and seeded in 96-well microtitration plates (5 x 10^3^ cells/well). The following day, infected cells were incubated with serial dilutions of plasma samples for 1 h before the addition of 5 x 10^3^ uninfected GFAB cells. After 72 h of infection, β-galactosidase expression by infected GFAB cells was detected by X-gal staining. Plasma samples from SFV-infected individuals BAD463, LOBAK2, BAD551 and BAK232 and one uninfected control (MEBAK189) were tested for the neutralization of BHK-21 (A, B) and HT1080 (C, D) cells infected with CI-PFV (A, C) or CII-SFV7 (B, D). Results are expressed as the infectivity relative to that of untreated cells and are presented as a function of the inverse of plasma sample dilution; the means and standard errors from triplicates are shown. The number of infectious units/well transmitted by untreated infected cells was 868 (CI-PFV infected-BHK-21, panel A), 436 (CII-SFV7 infected-BHK-21, panel F), 682 (CI-PFV infected-HT1080, panel C), and 591 (CII-SFV7 infected-HT1080, panel D). Panels E and F. Transmitter BHK-21 cells were infected at a moi of 0.05 with CI-PFV (E) or CII-SFV7 (F) for 72 h and seeded in 96-well microtitration plates (5 x 10^3^ cells/well). The following day, infected cells were incubated with plasma samples diluted 1:80 for 1 h before the addition of 5 x 10^3^ uninfected GFAB cells. After 72 h of infection, β-galactosidase expression by infected GFAB cells was detected by X-gal staining. Results are expressed as the infectivity relative to that of untreated cells. The number of infectious units/well transmitted by untreated infected cells was 868 IU/well for CI-PFV and 436 IU/well for CII-SFV7. Cell-free virus neutralization by the same plasma samples is shown for comparison (filled squares, infectious dose was ≈ 100 IU/ml according to the experimental design [17]). The relative infectivity of cell-transmitted virus in the presence of plasma samples is shown by open squares and labelled as “cells” on the x axis. The relative infectivity of cell-free virus in the presence of the same plasma sample is shown for comparison by filled squares, labelled as “virus” on the x axis. Data are shown for four plasma samples from uninfected controls (grey symbols), nine samples from SFV-infected individuals that neutralize the GI-D468 strain (blue symbols), five samples that neutralize the GII-K74 strain (red symbols) and four samples that neutralize both the GI-D468 and GII-K74 strains (purple symbols). *P* values from the paired t test are indicated in panels E and F.
(TIF)

S2 Fig Schematic description of the experiments. A: experiments presented in Fig 3; B: experiments presented in Figs 4–7; C: experiments presented in Fig 8; D: experiments presented in Fig 9A and 9B; E: experiments presented in Fig 9C and 9D; F: experiments presented in Figs 10, 11, S8–S10, and S1–S4 Movies. Figures were created with Biorender.com.
(PDF)

S3 Fig Quantitative analysis of fluorescent microscopy images of SFV-infected cultures. BHK-21 cells were infected at a moi of 0.05 with GI-D468, GII-K74, or CI-PFV in 25-cm^2^ flasks and seeded on glass coverslips (S2A Fig). Cells were cultured until the appearance of a cytopathic effect or for a maximum of three days. Cells were fixed with 2% PFA and stained with anti-SU-biotin+Streptavidin-AF488 and DAPI. Images were scanned at low magnification (10x). The STARDIST method was used for nuclei segmentation [51]. Four representative photographs (nuclei: blue, SU: green; scale bar = 100 μm) and results from the enumeration for three coverslips per viral strain are presented.
(TIF)

S4 Fig The anti-SU monoclonal antibody P3E10 fails to stain SFV ENV at the surface of infected cells, despite surface staining of sENV-GFP-transduced cells. A. BHK-21 cells were infected with GI-D468 or GII-K74 at a moi of 0.05, passed twice, and stained when a CPE was visible and when a significant percentage of permeabilized cells was labelled with anti-SU, as shown in Fig 8A. Here, cells were stained without permeabilization, and the staining was quantified by mean fluorescence intensity (mfi) of all cells. Mfi is presented on the x-axis and frequency expressed as the percentage of gated events on the y-axis on a histogram overlay. B. Cytometry analysis showing anti-SU staining of nonpermeabilized CI-PFV-GFP BHK cells; controls consisted of transfected cells stained with the secondary antibody only. The results are expressed as mfi_GFPpos_/mfi_GFPneg_ ratios. Representative dot-plots of gated single cells are shown. The ratios quantified in three independent experiments were 1.13, 1.25, and 1.55 C. Cytometry analysis showing anti-SU staining of nonpermeabilized BHK cells transduced with a plasmid encoding sENV-GFP (S7 Fig); controls consisted of transfected cells stained with the secondary antibody only. The results are expressed as mfi_GFPpos_/mfi_GFPneg_ ratios. Representative dot-plots of gated single cells are shown. The ratios quantified in four independent experiments were 3.1, 7.8, 4.5, and 5.1.
(TIF)

S5 Fig Binding of plasma samples to uninfected and CI-PFV-infected BHK-21 cells. Uninfected BHK-21 cells were incubated without plasma or with four human plasma samples diluted 1:10 in PBS-0.1% BSA. The specificity of the plasma samples is indicated in the legend. Staining was quantified by mean fluorescence intensity (mfi), as shown on the histogram overlay. Mfi are presented on the x-axis and frequency is expressed as the percentage of gated events on the y-axis. Nonspecific staining of mock cultures varied across plasma samples (panel A). Therefore, SFV-specific staining was quantified by the ratio of mfi of infected and mock cultures, as shown for CI-PFV-infected cells stained with the a-GI sample (panel B). CI-PFV^low^ and CI-PFV^high^ cells were infected for 6 days at a moi of 0.05 and 0.5, respectively.
(TIF)

S6 Fig The CI-PFV-GFP molecular clone. A. Schematic description: The overlapping *pol/env* gene is shown in black and highlighted by a box; the *gfp* gene is highlighted in green. Sequence modifications (red letters) in 3’ *pol* suppress ATG initiation in *env* and are silent in *pol*; the gfp codon-optimized coding sequence (green letters) is inserted one nucleotide after the *pol* stop codon to be in frame with *env*. It is followed by a sequence encoding a GS linker (blue letters) and the *env* gene. Silent mutations (red letters) in the beginning of *env* were inserted to avoid recombination between the duplicated *env/pol* overlapping sequence. B. Cytometry analysis showing co-expression of GFP with the Env SU subunit; controls consisted of unstained cells infected with CI-PFV-GFP. C. CI-PFV-GFP-infected BHK-21 cells were labelled with anti-LP-AF647 and DAPI; GFP and LP co-localized (yellow). GFP: green, LP: red, nuclei: blue; scale bars = 20 μm.
(TIF)

S7 Fig The modified sEnv-GFP protein. A. Schematic description: K15R, K34R, and K55R mutations in the LP cytoplasmic domain [48] and the truncation of the last 6 AA of the TM [39] were introduced into GI-D468 Env to prevent its intracellular retention and enhance its expression at the cell surface (red characters). The sequence of an Xba1 restriction site (encoding RSR) followed by the GFP sequence were fused at the C-term of TM (green characters). B. Cytometry analysis showing co-expression of GFP with the Env SU subunit in Triton-permeabilized BHK cells transduced with a plasmid encoding sENV-GFP; controls consisted of transfected cells stained with the secondary antibody only. Cells were permeabilized with Triton X-100. Data are expressed as the percentage of GFP^pos^ cells labelled with anti-SU among all viable cells C. sENV-GFP-transfected BHK-21 cells were labelled with anti-LP-AF647 and DAPI; GFP and LP co-localized. GFP: green, LP: red, nuclei: blue; scale bars = 20 μm.
(TIF)

S8 Fig CI-PFV-GFP cell-to-cell transmission visualized by live-cell imaging. CI-PFV-GFP infected HT1080 cells were mixed with uninfected cells to obtain a GFP^pos^ cell frequency of 5% and cultivated in 96-well plates (3000 cells/well) inside an IncuCyte device for 96 h (S2F Fig). Hourly acquisition was started within 30 min after seeding. We selected sequentially acquired images (one per hour) showing representative events in CI-PFV-GFP-infected cultures. A. Acquisition between 1D-11H and 1D-18H, 400 μm x 400 μm images with a superimposed 5 x 5 grid (80 μm between lines). Several GFP^bright^ cells established contact with uninfected cells. The contacts between the infected and uninfected cells lead to fusion (white triangles indicate cells that will be fused to the syncytium in the subsequent time frame) or not (blue triangles). B. Acquisition between 1D-5H and 1D-17H, 400 μm x 400 μm images with a superimposed 5 x 5 grid (80 μm between lines). The infected cells and syncytia display typically mesenchymal morphology. Their lamellipodium often split, leading to changes in direction. The uropod appears to remain anchored, either to the cells (white dots) or to the substratum (pink dots). The interaction with the substratum can generate structures that resemble intercellular connections, with elongated protrusions. Note that several GFP^neg^ cells are anchored to the substratum (yellow dots) or cells (blue dots).
(TIF)

S9 Fig Morphology and movement of BHK-21 uninfected cells. CI-PFV-GFP-infected BHK-21 cells were mixed with uninfected cells to obtain a frequency of 5% GFP-expressing cells and seeded at a density of 3000 cells/well in 96-well plates. Plates were cultivated in an IncuCyte Live-Cell Analysis device. Phase images were acquired every hour for four days using a 20x objective. The figure presents 250 μm x2 50 μm images acquired between 0D-01H and 0D-12H, with a superimposed 5 x 5 grid (50 μm between lines). For the first several hours, BHK cells start to adhere and spread on the surface. Once the cells acquire an orientation (0D 4H), they elongate and display a mesenchymal morphology. Some of the cells remain still, with limited motility detected at their edges (white and pink squares). Other cells start displaying migration and the direction is consistent over the observation period (blue squares). The migrating cells formed small clusters that moved in place (yellow square) or split (the two blue squares). Overall, at low cell density, the net displacement of uninfected BHK-21 cells was < 50 μm in 1 h and < 100–150 μm over 18 h.
(TIF)

S10 Fig Morphology and movement of HT1080 uninfected cells. CI-PFV-GFP-infected HT1080 cells were mixed with uninfected cells to obtain a frequency of 5% GFP-expressing cells and seeded at a density of 3000 cells/well in 96-well plates. Plates were cultivated in an IncuCyte Live-Cell Analysis device. Phase images were acquired every hour over four days using a 20x objective. The figure presents 400 μm x 400 μm images acquired between 0D-01H and 0D-12H, with a superimposed 5 x 5 grid (80 μm between lines). For the first several hours, HT1080 cells start to adhere and spread on the surface. Then, the cells acquire an orientation, elongate, and display typical fibroblast morphology, with lamellipodium leading the cell direction. The cells remain still and frequently change their orientation in the direction of neighboring cells (white square). Uropods adhere to adjacent cells (white dots) or to the substrate (pink dots). When reaching a cluster of cells, the incoming cell appears to insert itself into the nascent monolayer. Other cells leave the cluster to establish novel contacts (blue square). Overall, at low cell density, net displacement of uninfected HT1080 cells was < 100 μm over 12 h.
(TIF)

S1 Movie–BHK_SFV CI-PFV-GFP-infected BHK-21 cells were mixed with uninfected cells to obtain a frequency of 5% of GFP expressing cells and seeded at a density of 3000 cells/well in 96-well plates. Plates were cultivated in an IncuCyte Live-Cell Analysis device. Phase and green fluorescence photographs were acquired every hour over 4 days with a x4 objective and are presented at a 4 frames per second (fps).
(AVI)

S2 Movie–HT1080_SFV CI-PFV-GFP-infected HT1080 cells were mixed with uninfected cells to obtain a frequency of 5% of GFP expressing cells and seeded at a density of 3000 cells/well in 96-well plates. Plates were cultivated in an IncuCyte Live-Cell Analysis device and phase and green fluorescence photographs were acquired every hour over 4 days with a x4 objective and are presented at a 4 frames per second (fps).
(AVI)

S3 Movie–BHK_Mock Uninfected BHK-21 cells were seeded at a density of 3000 cells/well in 96-well plates. Plates were cultivated in an IncuCyte Live-Cell Analysis device and phase and green fluorescence photographs were acquired every hour over 4 days with a x4 objective and are presented at a 4 frames per second (fps).
(AVI)

S4 Movie–HT1080_Mock Uninfected HT1080 cells were seeded at a density of 3000 cells/well in 96-well plates. Plates were cultivated in an IncuCyte Live-Cell Analysis device and phase and green fluorescence photographs were acquired every hour over 4 days with a x4 objective and are presented at a 4 frames per second (fps).
(AVI)

## References

[1] Rethwilm A, Bodem J. Evolution of foamy viruses: the most ancient of all retroviruses. Viruses. 2013;5(10):2349–74. doi: 10.3390/v5102349 24072062 PMC3814592

[2] Gessain A, Rua R, Betsem E, Turpin J, Mahieux R. HTLV-3/4 and simian foamy retroviruses in humans: Discovery, epidemiology, cross-species transmission and molecular virology. Virology. 2013;435(1):187–99. doi: 10.1016/j.virol.2012.09.035 23217627 PMC7111966

[3] Pinto-Santini DM, Stenbak CR, Linial ML. Foamy virus zoonotic infections. Retrovirology. 2017;14:55. doi: 10.1186/s12977-017-0379-9 29197389 PMC5712078

[4] Rua R, Gessain A. Origin, evolution and innate immune control of simian foamy viruses in humans. Current Opinion in Virology. 2015;10:47–55. doi: 10.1016/j.coviro.2014.12.003 25698621 PMC7185842

[5] Boneva RS, Switzer WM, Spira TJ, Bhullar VB, Shanmugam V, Cong ME, et al. Clinical and virological characterization of persistent human infection with simian foamy viruses. AIDS Research and Human Retroviruses. 2007;23(11):1330–7. doi: 10.1089/aid.2007.0104 18184074

[6] Buseyne F, Betsem E, Montange T, Njouom R, Bilounga Ndongo C, Hermine O, et al. Clinical signs and blood test results among humans infected with zoonotic simian foamy virus: a case-control study. J Infect Dis. 2018;218(1):144–51. doi: 10.1093/infdis/jiy181 29608711

[7] Ledesma-Feliciano C, Troyer RM, Zheng X, Miller C, Cianciolo R, Bordicchia M, et al. Feline foamy virus infection: Characterization of experimental infection and prevalence of natural infection in domestic cats with and without chronic kidney disease. Viruses. 2019;11(7):662. 10.3390/v11070662 PMC6669521 31330990

[8] Gessain A, Montange T, Betsem E, Bilounga Ndongo C, Njouom R, Buseyne F. Case-control study of immune status in humans infected with zoonotic gorilla simian foamy viruses. J Infect Dis. 2020;221(10):1724–33. doi: 10.1093/infdis/jiz660 31822908

[9] Jones-Engel L, May CC, Engel GA, Steinkraus KA, Schillaci MA, Fuentes A, et al. Diverse contexts of zoonotic transmission of simian foamy viruses in Asia. Emerging Infectious Diseases. 2008;14(8):1200–8. doi: 10.3201/eid1408.071430 18680642 PMC2562341

[10] Mouinga-Ondeme A, Kazanji M. Simian foamy virus in non-human primates and cross-species transmission to humans in Gabon: an emerging zoonotic disease in central Africa? Viruses. 2013;5(6):1536–52. doi: 10.3390/v5061536 23783811 PMC3717720

[11] Santos AF, Cavalcante LTF, Muniz CP, Switzer WM, Soares MA. Simian Foamy Viruses in Central and South America: A New World of Discovery. Viruses. 2019;11(10):967. 10.3390/v11100967 PMC6832937 31635161

[12] Heneine W, Switzer WM, Sandstrom P, Brown J, Vedapuri S, Schable CA, et al. Identification of a Human Population Infected With Simian Foamy Viruses. Nature Medicine. 1998;4(4):403–7. doi: 10.1038/nm0498-403 9546784

[13] Switzer WM, Bhullar V, Shanmugam V, Cong ME, Parekh B, Lerche NW, et al. Frequent simian foamy virus infection in persons occupationally exposed to nonhuman primates. J Virol. 2004;78(6):2780–9. doi: 10.1128/jvi.78.6.2780-2789.2004 14990698 PMC353775

[14] Rogers NG, Basnight M, Gibbs CJ, Gajdusek DC. Latent viruses in chimpanzees with experimental kuru. Nature. 1967;216(5114):446–9. doi: 10.1038/216446a0 4964401

[15] Hooks JJ, Rogers NG, Gibbs CJ, Gajdusek DC, Cutchins EC, Lampert P. Characterization and distribution of 2 new foamy viruses isolated from chimpanzees. Archiv Fur Die Gesamte Virusforschung. 1972;38(1):38–55. doi: 10.1007/BF01241354 4626670

[16] Flower RLP, Wilcox GE, Cook RD, Ellis TM. Detection and prevalence of serotypes of feline syncitial spumaviruses. Arch Virol. 1985;83(1–2):53–63. doi: 10.1007/BF01310964 3918526

[17] Lambert C, Couteaudier M, Gouzil J, Richard L, Montange T, Betsem E, et al. Potent neutralizing antibodies in humans infected with zoonotic simian foamy viruses target conserved epitopes located in the dimorphic domain of the surface envelope protein. PLoS Pathog. 2018;14(10):e1007293. doi: 10.1371/journal.ppat.1007293 30296302 PMC6193739

[18] Zemba M, Alke A, Bodem J, Winkler IG, Flower RLP, Pfrepper KI, et al. Construction of infectious feline foamy virus genomes: Cat antisera do not cross-neutralize feline foamy virus chimera with serotype-specific env sequences. Virology. 2000;266(1):150–6. doi: 10.1006/viro.1999.0037 10612669

[19] Winkler IG, Flugel RM, Lochelt M, Flower RLP. Detection and molecular characterisation of feline foamy virus serotypes in naturally infected cats. Virology. 1998;247(2):144–51. doi: 10.1006/viro.1998.9232 9705907

[20] Phung HTT, Ikeda Y, Miyazawa T, Nakamura K, Mochizuki M, Izumiya Y, et al. Genetic analyses of feline foamy virus isolates from domestic and wild feline species in geographically distinct areas. Virus Research. 2001;76(2):171–81. doi: 10.1016/s0168-1702(01)00275-1 11410316

[21] Richard L, Rua R, Betsem E, Mouinga-Ondeme A, Kazanji M, Leroy E, et al. Cocirculation of Two env Molecular Variants, of Possible Recombinant Origin, in Gorilla and Chimpanzee Simian Foamy Virus Strains from Central Africa. J Virol. 2015;89(24):12480–91. doi: 10.1128/JVI.01798-15 26446599 PMC4665256

[22] Aiewsakun P, Richard L, Gessain A, Mouinga-Ondémé A, Afonso PV, Katzourakis A. Modular nature of simian foamy virus genomes and their evolutionary history. Virus evolution. 2019;5(2):vez032. doi: 10.1093/ve/vez032 31636999 PMC6795992

[23] Muhle M, Bleiholder A, Lochelt M, Denner J. Epitope mapping of the antibody response against the envelope proteins of the feline foamy virus. Viral Immunol. 2017;30:388–95. doi: 10.1089/vim.2016.0156 28355125

[24] Lambert C, Batalie D, Montange T, Betsem E, Mouinga-Ondeme A, Njouom R, et al. An immunodominant and conserved B-cell epitope in the envelope of simian foamy virus recognized by humans infected with zoonotic strains from apes. J Virol. 2019;93(11):e00068–19. doi: 10.1128/JVI.00068-19 30894477 PMC6532072

[25] Bao Q, Hipp M, Hugo A, Lei J, Liu Y, Kehl T, et al. In Vitro Evolution of Bovine Foamy Virus Variants with Enhanced Cell-Free Virus Titers and Transmission. Viruses. 2015;7(11):2907. doi: 10.3390/v7112907 26569290 PMC4664980

[26] Liebermann H, Riebe R. Isolation of bovine syncitial virus in Germany. Archiv Fur Experimentelle Veterinarmedizin. 1981;35(6):917–9. 7342910

[27] Bieniasz PD, Rethwilm A, Pitman R, Daniel MD, Chrystie I, McClure MO. A comparative study of higher primate foamy viruses, including a new virus from a gorilla. Virology. 1995;207(1):217–28. doi: 10.1006/viro.1995.1068 7871729

[28] Zhang S, Liu X, Liang Z, Bing T, Qiao W, Tan J. The Influence of Envelope C-Terminus Amino Acid Composition on the Ratio of Cell-Free to Cell-Cell Transmission for Bovine Foamy Virus. Viruses. 2019;11(2). doi: 10.3390/v11020130 30708993 PMC6410131

[29] Bao Q, Hotz-Wagenblatt A, Betts MJ, Hipp M, Hugo A, Pougialis G, et al. Shared and cell type-specific adaptation strategies of Gag and Env yield high titer bovine foamy virus variants. Infect Genet Evol. 2020:104287. doi: 10.1016/j.meegid.2020.104287 32179148

[30] Plochmann K, Horn A, Gschmack E, Armbruster N, Krieg J, Wiktorowicz T, et al. Heparan Sulfate Is an Attachment Factor for Foamy Virus Entry. J Virol. 2012;86(18):10028–35. doi: 10.1128/JVI.00051-12 22787203 PMC3446549

[31] Nasimuzzaman M, Persons DA. Cell Membrane-associated Heparan Sulfate Is a Receptor for Prototype Foamy Virus in Human, Monkey, and Rodent Cells. Mol Ther. 2012;20(6):1158–66. doi: 10.1038/mt.2012.41 22434139 PMC3369305

[32] Picard-Maureau M, Jarmy G, Berg A, Rethwilm A, Lindemann D. Foamy virus envelope glycoprotein-mediated entry involves a pH-dependent fusion process. J Virol. 2003;77(8):4722–30. doi: 10.1128/jvi.77.8.4722-4730.2003 12663779 PMC152125

[33] Stirnnagel K, Schupp D, Dupont A, Kudryavtsev V, Reh J, Mullers E, et al. Differential pH-dependent cellular uptake pathways among foamy viruses elucidated using dual-colored fluorescent particles. Retrovirology. 2012;9:71. doi: 10.1186/1742-4690-9-71 22935135 PMC3495412

[34] Goepfert PA, Shaw K, Wang G, Bansal A, Edwards BH, Mulligan MJ. An endoplasmic reticulum retrieval signal partitions human foamy virus maturation to intracytoplasmic membranes. J Virol. 1999;73(9):7210–7. doi: 10.1128/JVI.73.9.7210-7217.1999 10438808 PMC104245

[35] Stanke N, Stange A, Luftenegger D, Zentgraf H, Lindemann D. Ubiquitination of the prototype foamy virus envelope glycoprotein leader peptide regulates subviral particle release. J Virol. 2005;79(24):15074–83. doi: 10.1128/JVI.79.24.15074-15083.2005 16306578 PMC1316034

[36] Baldwin DN, Linial ML. The roles of Pol and Env in the assembly pathway of human foamy virus. J Virol. 1998;72(5):3658–65. doi: 10.1128/JVI.72.5.3658-3665.1998 9557646 PMC109586

[37] Fischer N, Heinkelein M, Lindemann D, Enssle J, Baum C, Werder E, et al. Foamy virus particle formation. J Virol. 1998;72(2):1610–5. doi: 10.1128/JVI.72.2.1610-1615.1998 9445065 PMC124643

[38] Yu SYF, Eastman SW, Linial ML. Foamy virus capsid assembly occurs at a pericentriolar region through a cytoplasmic targeting/retention signal in Gag. Traffic. 2006;7(8):966–77. doi: 10.1111/j.1600-0854.2006.00448.x 16749903 PMC7488586

[39] Pietschmann T, Zentgraf H, Rethwilm A, Lindemann D. An evolutionarily conserved positively charged amino acid in the putative membrane-spanning domain of the foamy virus envelope protein controls fusion activity. J Virol. 2000;74(10):4474–82. doi: 10.1128/jvi.74.10.4474-4482.2000 10775583 PMC111968

[40] Hooks JJ, Burns W, Hayashi K, Geis S, Notkins AL. Viral spread in presence of neutralizing antibody: mechanisms of persistence in Foamy virus infection. Infection and Immunity. 1976;14(5):1172–8. doi: 10.1128/iai.14.5.1172-1178.1976 185150 PMC415510

[41] Rua R, Betsem E, Calattini S, Saib A, Gessain A. Genetic characterization of simian foamy viruses infecting humans. J Virol. 2012;86(24):13350–9. doi: 10.1128/JVI.01715-12 23015714 PMC3503051

[42] Betsem E, Rua R, Tortevoye P, Froment A, Gessain A. Frequent and recent human acquisition of simian foamy viruses through apes’ bites in central Africa. PLoS Pathog. 2011;7(10):e1002306. doi: 10.1371/journal.ppat.1002306 22046126 PMC3203161

[43] Khan AS, Bodem J, Buseyne F, Gessain A, Johnson W, Kuhn JH, et al. Spumaretroviruses: Updated taxonomy and nomenclature. Virology. 2018;516:158–64. doi: 10.1016/j.virol.2017.12.035 29407373 PMC11318574

[44] Lambert C, Rua R, Gessain A, Buseyne F. A new sensitive indicator cell line reveals cross-transactivation of the viral LTR by gorilla and chimpanzee simian foamy viruses. Virology. 2016;496:219–26. doi: 10.1016/j.virol.2016.06.010 27348053

[45] Lochelt M, Zentgraf H, Flugel RM. Construction of an infectious DNA clone of the full-length Human Spumaretrovirus genome and mutagenesis of the bel1 gene. Virology. 1991;184(1):43–54. doi: 10.1016/0042-6822(91)90820-2 1651600

[46] Wagner TC, Bodem J. Sequence errors in foamy virus sequences in the GenBank database: resequencing of the prototypic foamy virus proviral plasmids. Arch Virol. 2016. doi: 10.1007/s00705-016-3206-z 28040837

[47] Lehmann-Che J, Giron ML, Delelis O, Lochelt M, Bittoun P, Tobaly-Tapiero J, et al. Protease-dependent uncoating of a complex retrovirus. J Virol. 2005;79(14):9244–53. doi: 10.1128/JVI.79.14.9244-9253.2005 15994819 PMC1168774

[48] Stange A, Luftenegger D, Reh J, Weissenhorn W, Lindemann D. Subviral Particle Release Determinants of Prototype Foamy Virus. J Virol. 2008;82(20):9858–69. doi: 10.1128/JVI.00949-08 18684814 PMC2566296

[49] Couteaudier M, Calzada-Fraile D, Montange T, Gessain A, Buseyne F. Inhibitors of the interferon response increase the replication of gorilla simian foamy viruses. Virology. 2020;541:25–31. doi: 10.1016/j.virol.2019.11.019 31826843

[50] Duda A, Luftenegger D, Pietschmann T, Lindemann D. Characterization of the prototype foamy virus envelope glycoprotein receptor-binding domain. J Virol. 2006;80(16):8158–67. doi: 10.1128/JVI.00460-06 16873272 PMC1563792

[51] Schmidt U, Weigert M, Broaddus C, Myers G. Cell Detection with Star-Convex Polygons. In: Frangi A. SJ, Davatzikos C., Alberola-López C., Fichtinger G., editor. Medical Image Computing and Computer Assisted Intervention–MICCAI 2018. 11071: Springer, S. A.; 2018.

[52] Otsu N. A Threshold Selection Method from Gray-Level Histograms. IEEE Transactions on Systems, Man, and Cybernetics. 1979;9(1):62–6.

[53] Khan AS, Sears JF, Muller J, Galvin TA, Shahabuddin M. Sensitive assays for isolation and detection of simian foamy retroviruses. Journal of Clinical Microbiology. 1999;37(8):2678–86. doi: 10.1128/JCM.37.8.2678-2686.1999 10405421 PMC85313

[54] Sattentau QJ. Cell-to-cell spread of retroviruses. Viruses. 2010;2(6):1306–21. doi: 10.3390/v2061306 21994681 PMC3185708

[55] Schiffner T, Sattentau QJ, Duncan CJA. Cell-to-cell spread of HIV-1 and evasion of neutralizing antibodies. Vaccine. 2013;31(49):5789–97. doi: 10.1016/j.vaccine.2013.10.020 24140477

[56] Tiwari V, Koganti R, Russell G, Sharma A, Shukla D. Role of Tunneling Nanotubes in Viral Infection, Neurodegenerative Disease, and Cancer. Front Immunol. 2021;12:680891. doi: 10.3389/fimmu.2021.680891 34194434 PMC8236699

[57] Su B, Moog C. Which Antibody Functions are Important for an HIV Vaccine? Front Immunol. 2014;5:289. doi: 10.3389/fimmu.2014.00289 24995008 PMC4062070

[58] Dufloo J, Bruel T, Schwartz O. HIV-1 cell-to-cell transmission and broadly neutralizing antibodies. Retrovirology. 2018;15(1):51. doi: 10.1186/s12977-018-0434-1 30055632 PMC6064125

[59] Jolly C. Cell-to-cell transmission of retroviruses: Innate immunity and interferon-induced restriction factors. Virology. 2011;411(2):251–9. doi: 10.1016/j.virol.2010.12.031 21247613 PMC3053447

[60] Jakobsdottir GM, Iliopoulou M, Nolan R, Alvarez L, Compton AA, Padilla-Parra S. On the Whereabouts of HIV-1 Cellular Entry and Its Fusion Ports. Trends in Molecular Medicine. 2017;23(10):932–44. doi: 10.1016/j.molmed.2017.08.005 28899754

[61] Forthal DN, Finzi A. Antibody-dependent cellular cytotoxicity in HIV infection. AIDS. 2018;32(17):2439–51. doi: 10.1097/QAD.0000000000002011 30234611 PMC6497078

[62] Heinkelein M, Rammling M, Juretzek T, Lindemann D, Rethwilm A. Retrotransposition and cell-to-cell transfer of foamy viruses. J Virol. 2003;77(21):11855–8. doi: 10.1128/jvi.77.21.11855-11858.2003 14557671 PMC229254

[63] Anand SP, Finzi A. Understudied Factors Influencing Fc-Mediated Immune Responses against Viral Infections. Vaccines. 2019;7(3). doi: 10.3390/vaccines7030103 31480293 PMC6789852

[64] Lindemann D, Pietschmann T, Picard-Maureau M, Berg A, Heinkelein M, Thurow J, et al. A particle-associated glycoprotein signal peptide essential for virus maturation and infectivity. J Virol. 2001;75(13):5762–71. doi: 10.1128/JVI.75.13.5762-5771.2001 11390578 PMC114292

[65] Clayton KL, Mylvaganam G, Villasmil-Ocando A, Stuart H, Maus MV, Rashidian M, et al. HIV-infected macrophages resist efficient NK cell-mediated killing while preserving inflammatory cytokine responses. Cell Host Mic. 2021;29(3):435–47.e9. doi: 10.1016/j.chom.2021.01.006 33571449 PMC8486985

[66] Jansens RJJ, Tishchenko A, Favoreel HW. Bridging the Gap: Virus Long-Distance Spread via Tunneling Nanotubes. J Virol. 2020;94(8). doi: 10.1128/JVI.02120-19 32024778 PMC7108841

[67] Baba E, Nakamura M, Tanaka Y, Kuroki M, Itoyama Y, Nakano S, et al. Multiple neutralizing B-cell epitopes of human T-cell leukemia virus type 1 (HTLV-1) identified by human monoclonal antibodies. J Immunol. 1993;151(2):1013–24. 7687611

[68] Desgranges C, Souche S, Vernant JC, Smadja D, Vahlne A, Horal P. Identification of novel neutralization-inducing regions of the human T-cell lymphotropic virus type I envelope glycoproteins with human HTLV-1-seropositive sera. Aids Research and Human Retroviruses. 1994;10(2):163–73. doi: 10.1089/aid.1994.10.163 8198868

[69] Mizuguchi M, Takahashi Y, Tanaka R, Fukushima T, Tanaka Y. Conservation of a Neutralization Epitope of Human T-cell Leukemia Virus Type 1 (HTLV-1) among Currently Endemic Clinical Isolates in Okinawa, Japan. Pathogens. 2020;9(2). doi: 10.3390/pathogens9020082 32012672 PMC7168584

[70] Gurke S, Barroso JF, Gerdes HH. The art of cellular communication: tunneling nanotubes bridge the divide. Histochemistry and cell biology. 2008;129(5):539–50. doi: 10.1007/s00418-008-0412-0 18386044 PMC2323029

[71] Abounit S, Zurzolo C. Wiring through tunneling nanotubes—from electrical signals to organelle transfer. Journal of Cell Science. 2012;125(5):1089–98. doi: 10.1242/jcs.083279 22399801

[72] Chang K, Baginski J, Hassan SF, Volin M, Shukla D, Tiwari V. Filopodia and Viruses: An Analysis of Membrane Processes in Entry Mechanisms. Front Microbiol. 2016;7:300. doi: 10.3389/fmicb.2016.00300 27014223 PMC4785137

[73] Oh MJ, Akhtar J, Desai P, Shukla D. A role for heparan sulfate in viral surfing. Biochem Biophys Res Commun. 2010;391(1):176–81. doi: 10.1016/j.bbrc.2009.11.027 19909728 PMC2812628

[74] Sherer NM, Lehmann MJ, Jimenez-Soto LF, Horensavitz C, Pypaert M, Mothes W. Retroviruses can establish filopodial bridges for efficient cell-to-cell transmission. Nature Cell Biology. 2007;9(3):310–U106. doi: 10.1038/ncb1544 17293854 PMC2628976

[75] Sowinski S, Jolly C, Berninghausen O, Purbhoo MA, Chauveau A, Kohler K, et al. Membrane nanotubes physically connect T cells over long distances presenting a novel route for HIV-1 transmission. Nat Cell Biol. 2008;10(2):211–9. doi: 10.1038/ncb1682 18193035

[76] Van Prooyen N, Gold H, Andresen V, Schwartz O, Jones K, Ruscetti F, et al. Human T-cell leukemia virus type 1 p8 protein increases cellular conduits and virus transmission. PNAS. 2010;107(48):20738–43. doi: 10.1073/pnas.1009635107 21076035 PMC2996430

[77] Omsland M, Pise-Masison C, Fujikawa D, Galli V, Fenizia C, Parks RW, et al. Inhibition of Tunneling Nanotube (TNT) Formation and Human T-cell Leukemia Virus Type 1 (HTLV-1) Transmission by Cytarabine. Sci Rep. 2018;8(1):11118. doi: 10.1038/s41598-018-29391-w 30042514 PMC6057998

[78] Shimauchi T, Caucheteux S, Finsterbusch K, Turpin J, Blanchet F, Ladell K, et al. Dendritic cells promote the spread of Human T-Cell Leukemia Virus type 1 via bidirectional interactions with CD4+ T cells. J Invest Dermatol. 2019;139(1):157–66. doi: 10.1016/j.jid.2018.06.188 30048652

[79] Donhauser N, Socher E, Millen S, Heym S, Sticht H, Thoma-Kress AK. Transfer of HTLV-1 p8 and Gag to target T-cells depends on VASP, a novel interaction partner of p8. PLoS Pathog. 2020;16(9):e1008879. doi: 10.1371/journal.ppat.1008879 32997728 PMC7526893

[80] Budzik KM, Nace RA, Ikeda Y, Russell SJ. Oncolytic Foamy Virus: Generation and Properties of a Nonpathogenic Replicating Retroviral Vector System That Targets Chronically Proliferating Cancer Cells. J Virol. 2021;95(10):e00015–21. doi: 10.1128/JVI.00015-21 33692205 PMC8139661

[81] Mouquet H. Antibody B cell responses in HIV-1 infection. Trends Immunol. 2014;35(11):549–61. doi: 10.1016/j.it.2014.08.007 25240985

[82] Su B, Dispinseri S, Iannone V, Zhang T, Wu H, Carapito R, et al. Update on Fc-Mediated Antibody Functions Against HIV-1 Beyond Neutralization. Front Immunol. 2019;10. 31921207 10.3389/fimmu.2019.02968 PMC6930241

[83] Williams DK, Khan AS. Role of neutralizing antibodies in controlling simian foamy virus transmission and infection. Transfusion. 2010;50(1):200–7. doi: 10.1111/j.1537-2995.2009.02372.x 19719470

[84] Bruel T, Guivel-Benhassine F, Amraoui S, Malbec M, Richard L, Bourdic K, et al. Elimination of HIV-1-infected cells by broadly neutralizing antibodies. Nat Commun. 2016;7. doi: 10.1038/ncomms10844 26936020 PMC4782064

[85] Richard J, Prevost J, Alsahafi N, Ding SL, Finzi A. Impact of HIV-1 Envelope Conformation on ADCC Responses. Trends in Microbiology. 2018;26(4):253–65. doi: 10.1016/j.tim.2017.10.007 29162391

[86] Pietschmann T, Heinkelein M, Heldmann M, Zentgraf H, Rethwilm A, Lindemann D. Foamy virus capsids require the cognate envelope protein for particle export. J Virol. 1999;73(4):2613–21. doi: 10.1128/JVI.73.4.2613-2621.1999 10074106 PMC104016

