# Supplementary material for: Plasma antibodies from humans infected with zoonotic simian foamy virus do not inhibit cell-to-cell transmission of the virus despite binding to the surface of infected cells
Source: PLoS Pathog. 2022 May 23;18(5):e1010470. doi: 10.1371/journal.ppat.1010470 (PMC9166401; doi:10.1371/journal.ppat.1010470)
Supplement: S1 Table — (DOCX) [file ppat.1010470.s001.docx]

**Table S1. Demographics and SFV infection status of individuals whose plasma samples were tested in the study.**

| Code | Ethnicity | Age (yrs) | Estimated duration of infection (yrs) | SFV infection status | Neutralized genotype |
| --- | --- | --- | --- | --- | --- |
| BAD356 | Bantu | 60 |  | Neg |  |
| BAK141 | Pygmy | 62 |  | Neg |  |
| BAK181 | Pygmy | 39 |  | Neg |  |
| BAK183 | Pygmy | 41 |  | Neg |  |
| BAK231 | Pygmy | 50 |  | Neg |  |
| BAK227 | Pygmy | 59 |  | Neg |  |
| BAK301 | Pygmy | 39 |  | Neg |  |
| MEBAK189 | Pygmy | 52 |  | Neg |  |
| MEBAK195 | Pygmy | 48 |  | Neg |  |
| BAD332 | Bantu | 37 | 12 | Pos | GI |
| BAD348 | Bantu | 33 | 14 | Pos | GI |
| BAD350 | Bantu | 68 | 28 | Pos | GII |
| BAD447 | Bantu | 59 | 19 | Pos | GI+GII |
| BAD456 | Bantu | 35 | 13 | Pos | GI |
| BAD463 | Bantu | 46 | 9 | Pos | GI |
| BAD468 | Bantu | 38 | 15 | Pos | GI+GII |
| BAD551 | Bantu | 41 | 14 | Pos | GII |
| BAK56 | Pygmy | 75 | 35 | Pos | GI |
| BAK74 | Pygmy | 50 | 24 | Pos | GI+GII |
| BAK132 | Pygmy | 69 | 39 | Pos | GI |
| BAK133 | Pygmy | 59 | 29 | Pos | GII |
| BAK228 | Pygmy | 70 | 40 | Pos | GII |
| BAK232 | Pygmy | 62 | 22 | Pos | GII |
| BOBAK153 | Pygmy | 68 | 15 | Pos | GI |
| H9GAB49 | Bantu | 58 | 25 | Pos | GI |
| H12GAB69 | Bantu | 38 | 16 | Pos | GI+GII |
| LOBAK2 | Pygmy | 83 | 53 | Pos | GI |
| MEBAK65 | Pygmy | 40 | 20 | Pos | GI |
| MEBAK88 | Pygmy | 64 | 19 | Pos | GII |
