# Supplementary figures and images for: Plasma antibodies from humans infected with zoonotic simian foamy virus do not inhibit cell-to-cell transmission of the virus despite binding to the surface of infected cells
Source: PLoS Pathog. 2022 May 23;18(5):e1010470. doi: 10.1371/journal.ppat.1010470 (PMC9166401; doi:10.1371/journal.ppat.1010470)

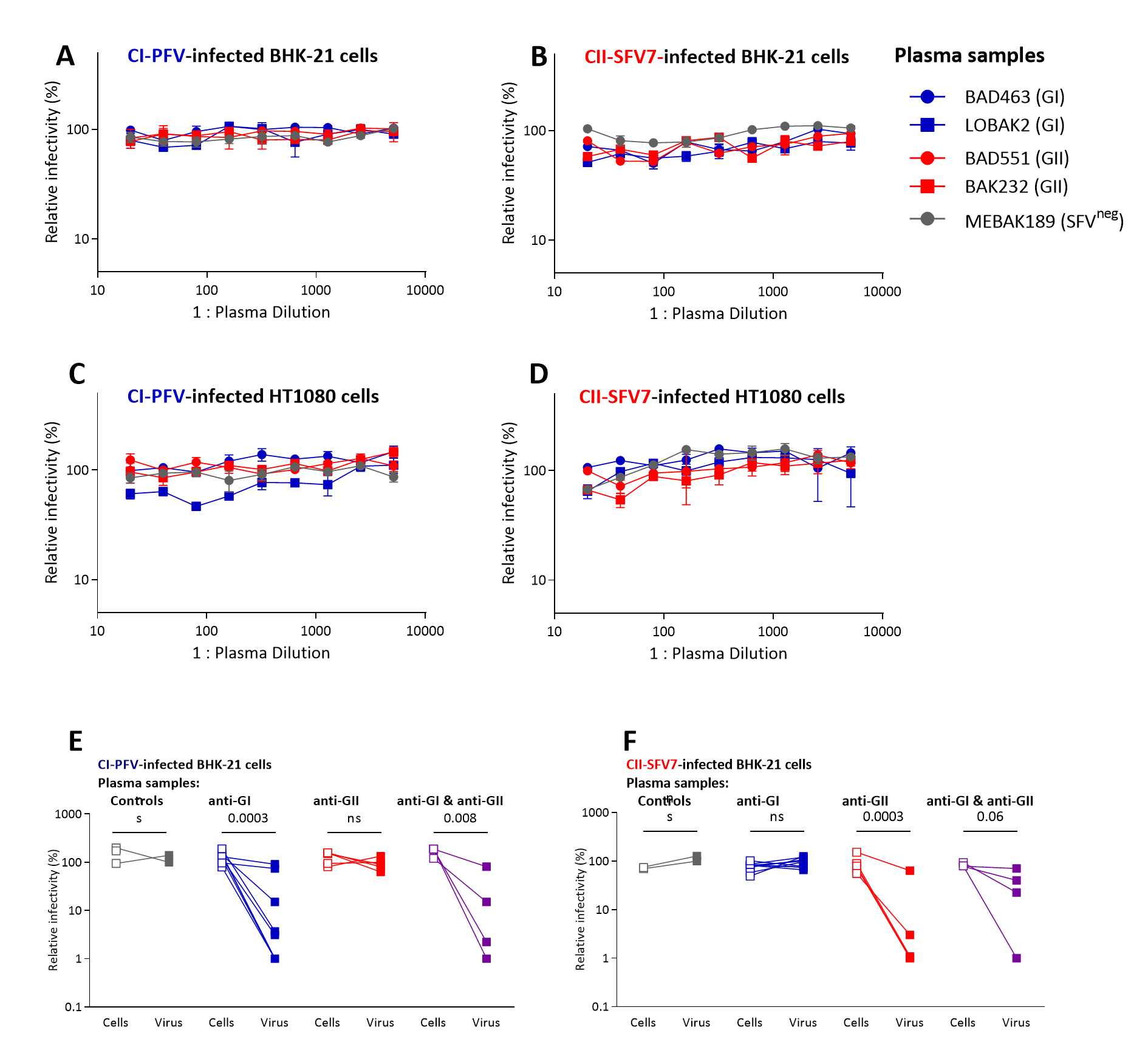

Supplement: S1 Fig — Experiments were performed as described in Fig 2. Transmitter cells were infected at a moi of 0.05 for 72 h and seeded in 96-well microtitration plates (5 x 10^3^ cells/well). The following day, infected cells were incubated with serial dilutions of plasma samples for 1 h before the addition of 5 x 10^3^ uninfected GFAB cells. After 72 h of infection, β-galactosidase expression by infected GFAB cells was detected by X-gal staining. Plasma samples from SFV-infected individuals BAD463, LOBAK2, BAD551 and BAK232 and one uninfected control (MEBAK189) were tested for the neutralization of BHK-21 (A, B) and HT1080 (C, D) cells infected with CI-PFV (A, C) or CII-SFV7 (B, D). Results are expressed as the infectivity relative to that of untreated cells and are presented as a function of the inverse of plasma sample dilution; the means and standard errors from triplicates are shown. The number of infectious units/well transmitted by untreated infected cells was 868 (CI-PFV infected-BHK-21, panel A), 436 (CII-SFV7 infected-BHK-21, panel F), 682 (CI-PFV infected-HT1080, panel C), and 591 (CII-SFV7 infected-HT1080, panel D). Panels E and F. Transmitter BHK-21 cells were infected at a moi of 0.05 with CI-PFV (E) or CII-SFV7 (F) for 72 h and seeded in 96-well microtitration plates (5 x 10^3^ cells/well). The following day, infected cells were incubated with plasma samples diluted 1:80 for 1 h before the addition of 5 x 10^3^ uninfected GFAB cells. After 72 h of infection, β-galactosidase expression by infected GFAB cells was detected by X-gal staining. Results are expressed as the infectivity relative to that of untreated cells. The number of infectious units/well transmitted by untreated infected cells was 868 IU/well for CI-PFV and 436 IU/well for CII-SFV7. Cell-free virus neutralization by the same plasma samples is shown for comparison (filled squares, infectious dose was ≈ 100 IU/ml according to the experimental design [17]). The relative infectivity of cell-transmitted virus in the [file ppat.1010470.s002.tif]

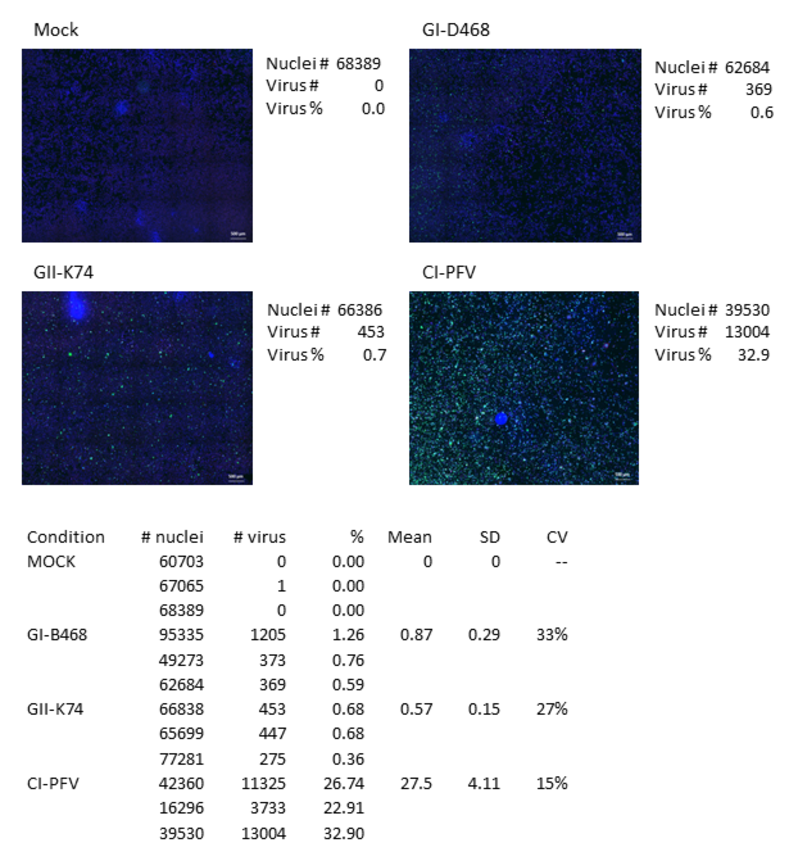

Supplement: S3 Fig — BHK-21 cells were infected at a moi of 0.05 with GI-D468, GII-K74, or CI-PFV in 25-cm2 flasks and seeded on glass coverslips (S2A Fig). Cells were cultured until the appearance of a cytopathic effect or for a maximum of three days. Cells were fixed with 2% PFA and stained with anti-SU-biotin+Streptavidin-AF488 and DAPI. Images were scanned at low magnification (10x). The STARDIST method was used for nuclei segmentation [51]. Four representative photographs (nuclei: blue, SU: green; scale bar = 100 μm) and results from the enumeration for three coverslips per viral strain are presented. (TIF) [file ppat.1010470.s004.tif]

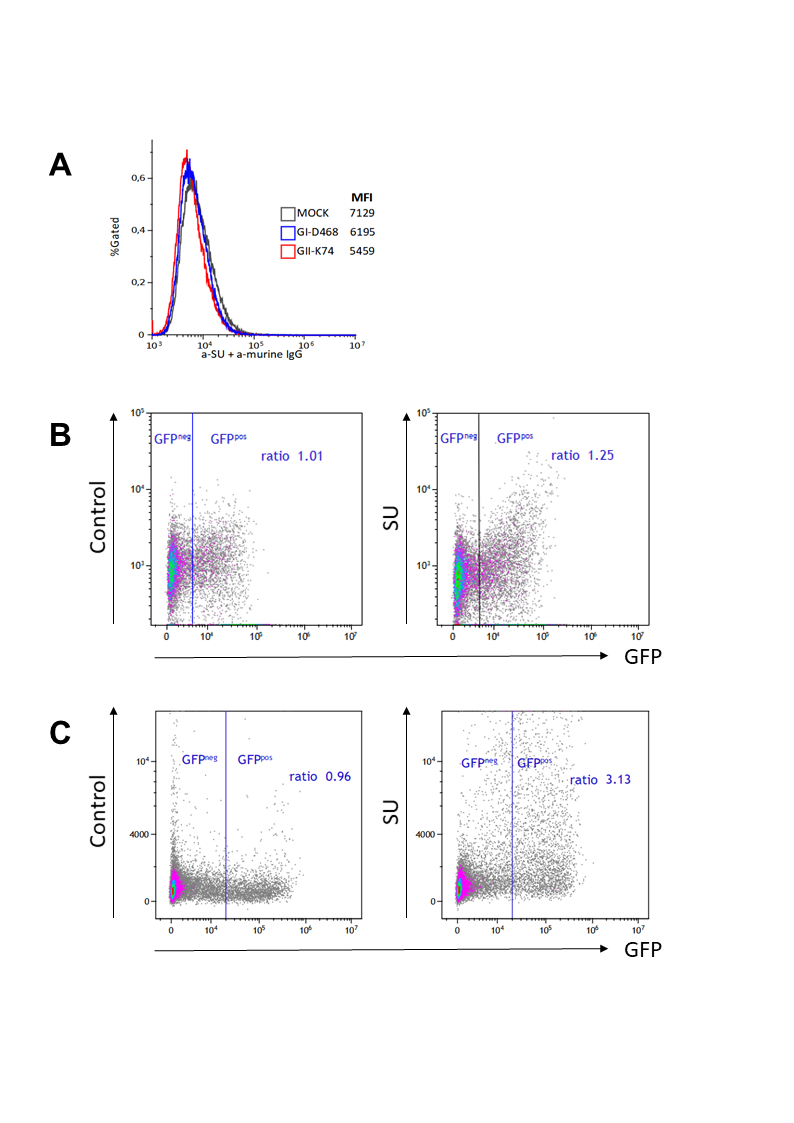

Supplement: S4 Fig — A. BHK-21 cells were infected with GI-D468 or GII-K74 at a moi of 0.05, passed twice, and stained when a CPE was visible and when a significant percentage of permeabilized cells was labelled with anti-SU, as shown in Fig 8A. Here, cells were stained without permeabilization, and the staining was quantified by mean fluorescence intensity (mfi) of all cells. Mfi is presented on the x-axis and frequency expressed as the percentage of gated events on the y-axis on a histogram overlay. B. Cytometry analysis showing anti-SU staining of nonpermeabilized CI-PFV-GFP BHK cells; controls consisted of transfected cells stained with the secondary antibody only. The results are expressed as mfiGFPpos/mfiGFPneg ratios. Representative dot-plots of gated single cells are shown. The ratios quantified in three independent experiments were 1.13, 1.25, and 1.55 C. Cytometry analysis showing anti-SU staining of nonpermeabilized BHK cells transduced with a plasmid encoding sENV-GFP (S7 Fig); controls consisted of transfected cells stained with the secondary antibody only. The results are expressed as mfiGFPpos/mfiGFPneg ratios. Representative dot-plots of gated single cells are shown. The ratios quantified in four independent experiments were 3.1, 7.8, 4.5, and 5.1. (TIF) [file ppat.1010470.s005.tif]

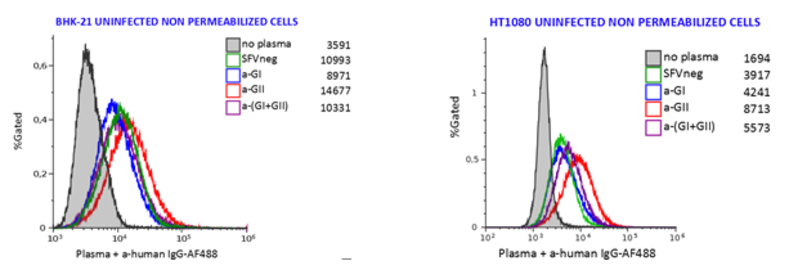

Supplement: S5 Fig — Uninfected BHK-21 cells were incubated without plasma or with four human plasma samples diluted 1:10 in PBS-0.1% BSA. The specificity of the plasma samples is indicated in the legend. Staining was quantified by mean fluorescence intensity (mfi), as shown on the histogram overlay. Mfi are presented on the x-axis and frequency is expressed as the percentage of gated events on the y-axis. Nonspecific staining of mock cultures varied across plasma samples (panel A). Therefore, SFV-specific staining was quantified by the ratio of mfi of infected and mock cultures, as shown for CI-PFV-infected cells stained with the a-GI sample (panel B). CI-PFVlow and CI-PFVhigh cells were infected for 6 days at a moi of 0.05 and 0.5, respectively. (TIF) [file ppat.1010470.s006.tif]

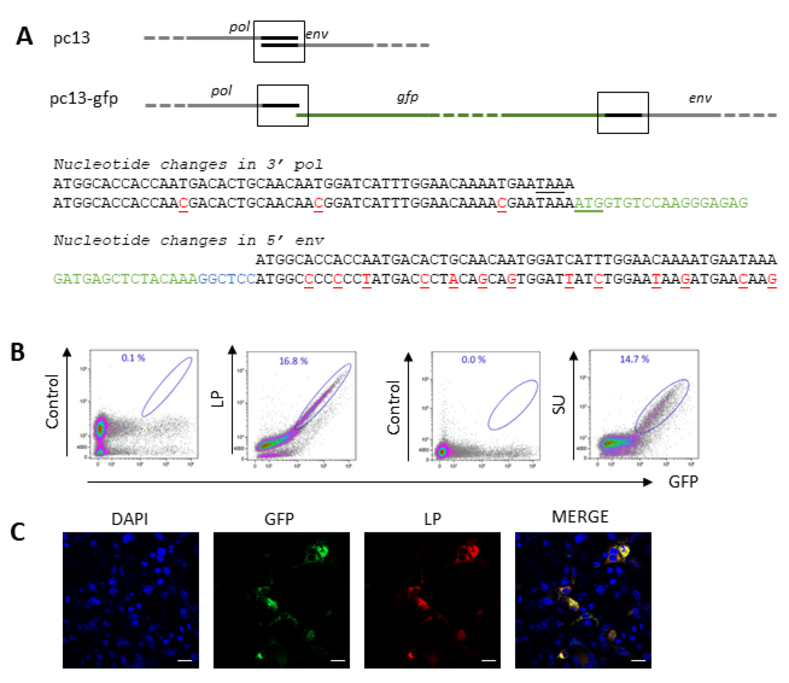

Supplement: S6 Fig — A. Schematic description: The overlapping pol/env gene is shown in black and highlighted by a box; the gfp gene is highlighted in green. Sequence modifications (red letters) in 3’ pol suppress ATG initiation in env and are silent in pol; the gfp codon-optimized coding sequence (green letters) is inserted one nucleotide after the pol stop codon to be in frame with env. It is followed by a sequence encoding a GS linker (blue letters) and the env gene. Silent mutations (red letters) in the beginning of env were inserted to avoid recombination between the duplicated env/pol overlapping sequence. B. Cytometry analysis showing co-expression of GFP with the Env SU subunit; controls consisted of unstained cells infected with CI-PFV-GFP. C. CI-PFV-GFP-infected BHK-21 cells were labelled with anti-LP-AF647 and DAPI; GFP and LP co-localized (yellow). GFP: green, LP: red, nuclei: blue; scale bars = 20 μm. (TIF) [file ppat.1010470.s007.tif]

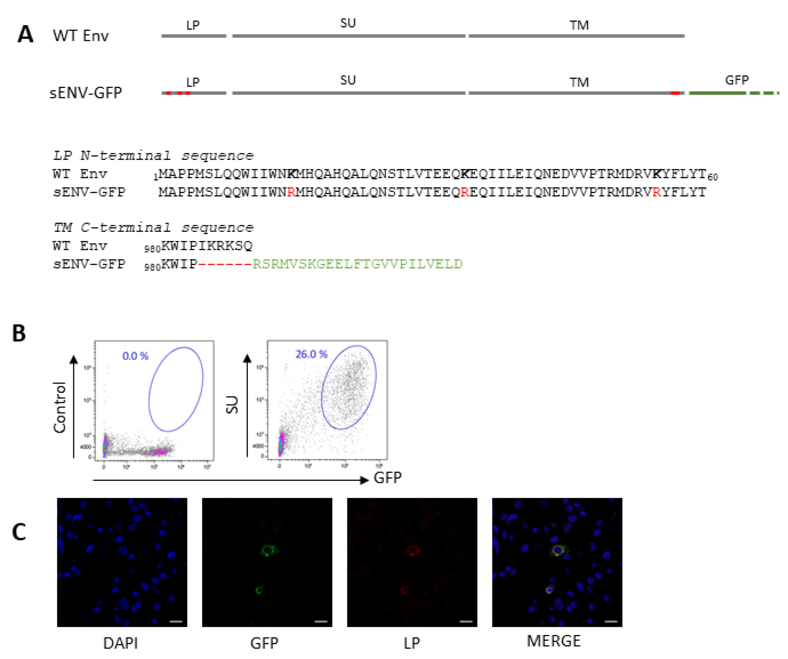

Supplement: S7 Fig — A. Schematic description: K15R, K34R, and K55R mutations in the LP cytoplasmic domain [48] and the truncation of the last 6 AA of the TM [39] were introduced into GI-D468 Env to prevent its intracellular retention and enhance its expression at the cell surface (red characters). The sequence of an Xba1 restriction site (encoding RSR) followed by the GFP sequence were fused at the C-term of TM (green characters). B. Cytometry analysis showing co-expression of GFP with the Env SU subunit in Triton-permeabilized BHK cells transduced with a plasmid encoding sENV-GFP; controls consisted of transfected cells stained with the secondary antibody only. Cells were permeabilized with Triton X-100. Data are expressed as the percentage of GFPpos cells labelled with anti-SU among all viable cells C. sENV-GFP-transfected BHK-21 cells were labelled with anti-LP-AF647 and DAPI; GFP and LP co-localized. GFP: green, LP: red, nuclei: blue; scale bars = 20 μm. (TIF) [file ppat.1010470.s008.tif]

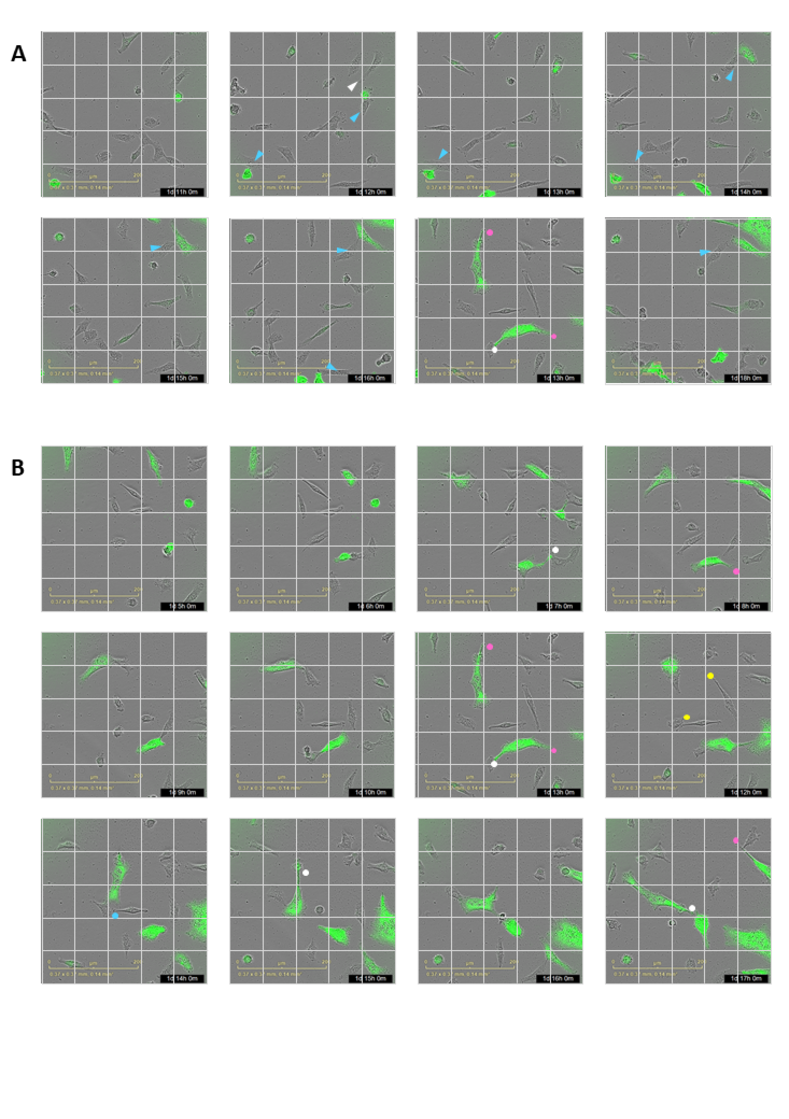

Supplement: S8 Fig — CI-PFV-GFP infected HT1080 cells were mixed with uninfected cells to obtain a GFPpos cell frequency of 5% and cultivated in 96-well plates (3000 cells/well) inside an IncuCyte device for 96 h (S2F Fig). Hourly acquisition was started within 30 min after seeding. We selected sequentially acquired images (one per hour) showing representative events in CI-PFV-GFP-infected cultures. A. Acquisition between 1D-11H and 1D-18H, 400 μm x 400 μm images with a superimposed 5 x 5 grid (80 μm between lines). Several GFPbright cells established contact with uninfected cells. The contacts between the infected and uninfected cells lead to fusion (white triangles indicate cells that will be fused to the syncytium in the subsequent time frame) or not (blue triangles). B. Acquisition between 1D-5H and 1D-17H, 400 μm x 400 μm images with a superimposed 5 x 5 grid (80 μm between lines). The infected cells and syncytia display typically mesenchymal morphology. Their lamellipodium often split, leading to changes in direction. The uropod appears to remain anchored, either to the cells (white dots) or to the substratum (pink dots). The interaction with the substratum can generate structures that resemble intercellular connections, with elongated protrusions. Note that several GFPneg cells are anchored to the substratum (yellow dots) or cells (blue dots). (TIF) [file ppat.1010470.s009.tif]

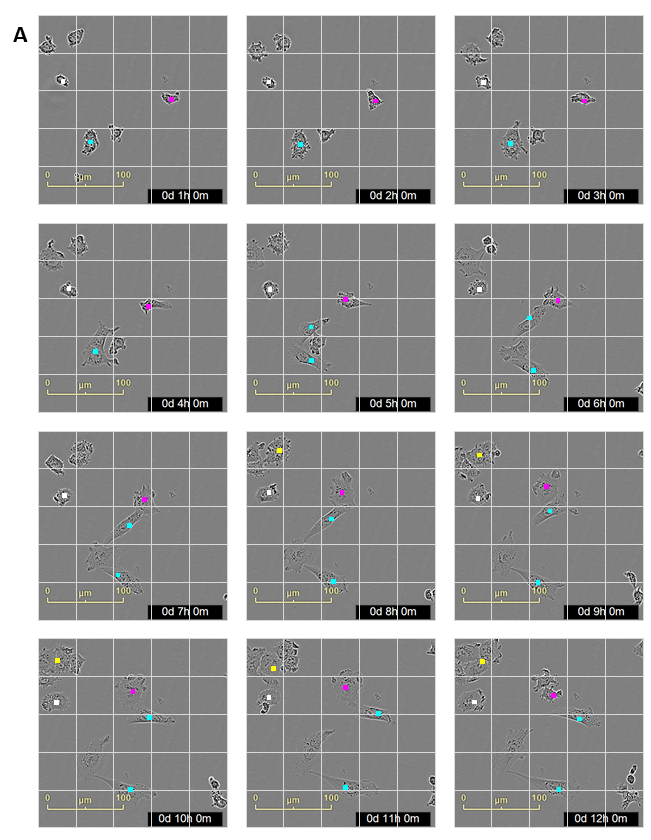

Supplement: S9 Fig — CI-PFV-GFP-infected BHK-21 cells were mixed with uninfected cells to obtain a frequency of 5% GFP-expressing cells and seeded at a density of 3000 cells/well in 96-well plates. Plates were cultivated in an IncuCyte Live-Cell Analysis device. Phase images were acquired every hour for four days using a 20x objective. The figure presents 250 μm x2 50 μm images acquired between 0D-01H and 0D-12H, with a superimposed 5 x 5 grid (50 μm between lines). For the first several hours, BHK cells start to adhere and spread on the surface. Once the cells acquire an orientation (0D 4H), they elongate and display a mesenchymal morphology. Some of the cells remain still, with limited motility detected at their edges (white and pink squares). Other cells start displaying migration and the direction is consistent over the observation period (blue squares). The migrating cells formed small clusters that moved in place (yellow square) or split (the two blue squares). Overall, at low cell density, the net displacement of uninfected BHK-21 cells was < 50 μm in 1 h and < 100–150 μm over 18 h. (TIF) [file ppat.1010470.s010.tif]

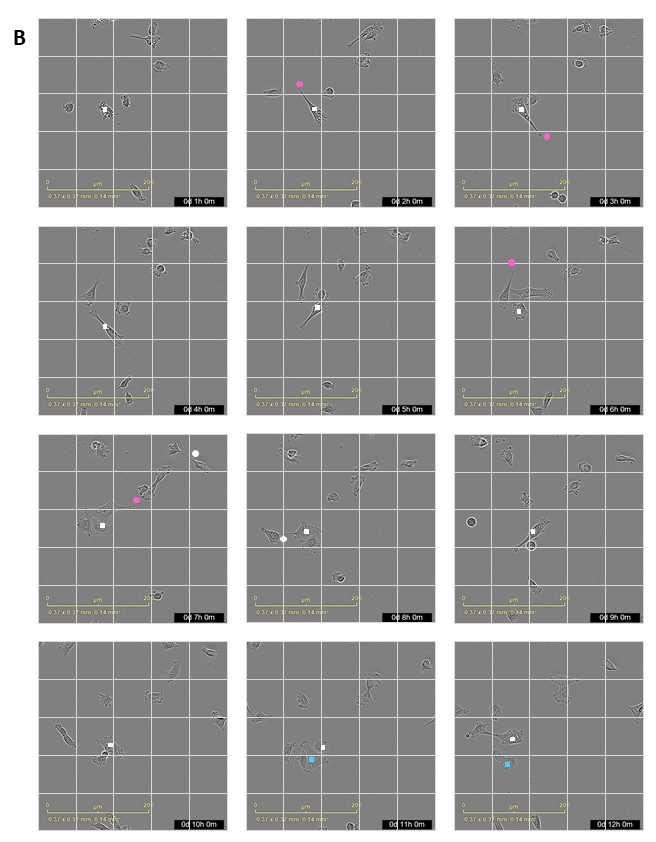

Supplement: S10 Fig — CI-PFV-GFP-infected HT1080 cells were mixed with uninfected cells to obtain a frequency of 5% GFP-expressing cells and seeded at a density of 3000 cells/well in 96-well plates. Plates were cultivated in an IncuCyte Live-Cell Analysis device. Phase images were acquired every hour over four days using a 20x objective. The figure presents 400 μm x 400 μm images acquired between 0D-01H and 0D-12H, with a superimposed 5 x 5 grid (80 μm between lines). For the first several hours, HT1080 cells start to adhere and spread on the surface. Then, the cells acquire an orientation, elongate, and display typical fibroblast morphology, with lamellipodium leading the cell direction. The cells remain still and frequently change their orientation in the direction of neighboring cells (white square). Uropods adhere to adjacent cells (white dots) or to the substrate (pink dots). When reaching a cluster of cells, the incoming cell appears to insert itself into the nascent monolayer. Other cells leave the cluster to establish novel contacts (blue square). Overall, at low cell density, net displacement of uninfected HT1080 cells was < 100 μm over 12 h. (TIF) [file ppat.1010470.s011.tif]
