# Supplementary material for: Plasma antibodies from humans infected with zoonotic simian foamy virus do not inhibit cell-to-cell transmission of the virus despite binding to the surface of infected cells
Source: PLoS Pathog. 2022 May 23;18(5):e1010470. doi: 10.1371/journal.ppat.1010470 (PMC9166401; doi:10.1371/journal.ppat.1010470)
Supplement: S2 Fig — A: experiments presented in Fig 3; B: experiments presented in Figs 4–7; C: experiments presented in Fig 8; D: experiments presented in Fig 9A and 9B; E: experiments presented in Fig 9C and 9D; F: experiments presented in Figs 10, 11, S8–S10, and S1–S4 Movies. Figures were created with Biorender.com. (PDF) [file ppat.1010470.s003.pdf]

**S2A Fig. Schematic description of experiments presented in Fig 3**

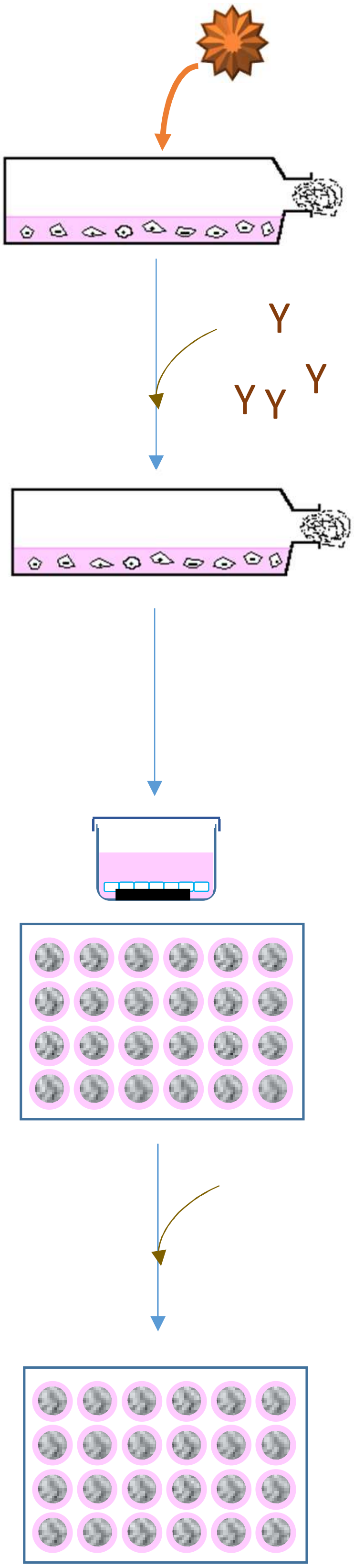

## S2B Fig. Schematic description of experiments presented in Figs 4-7

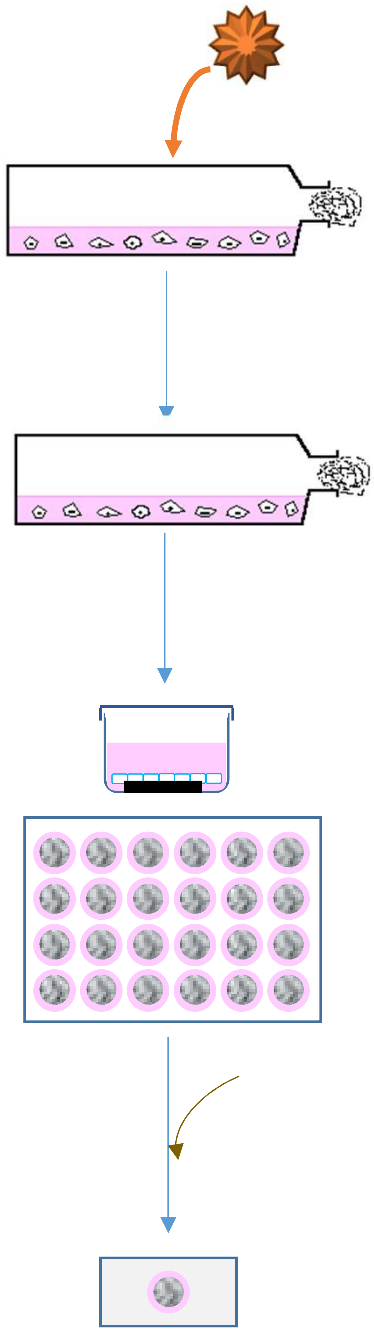

Infection of susceptible cells with SFV in 25 $cm^2$ flask, moi=0.05

+ 72 hrs
Pass cells

+ 72 hrs
Seed cells in 24-well plates containing glass coverslips

Daily examination, fixation between ECP appearance and full cell lysis (+24 hrs to + 72 hrs)

+ PFA 2%

Storage at +4°C
Staining of cells on coverslips + Microscopy analysis

| Figure | Cell permeabilization with Triton | SFV staining |
|---|---|---|
| 4 & 5 | Yes | MoAb anti-SU |
| 6 | No & Yes | Plasma from SFV-infected and uninfected individuals |
| 7 | No | Plasma from SFV-infected individuals |

**S2C Fig. Schematic description of experiments presented in Figs 8**

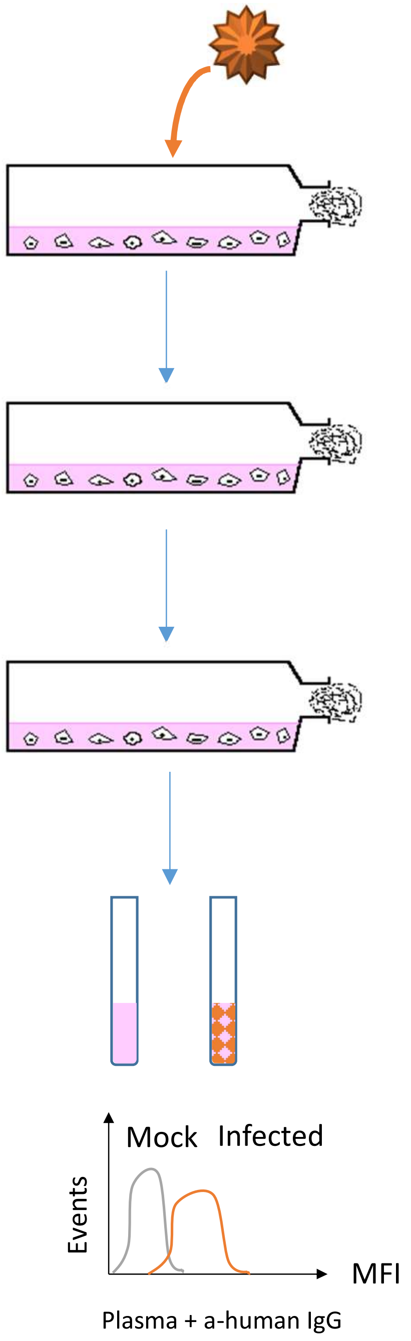


Infection of BHK-21 cells with SFV in 25 $cm^2$ flask, moi=0.05

+ 72 hrs
Pass cells

+ 72 hrs
Pass cells

+ 24 hrs to + 72 hrs (according to CPE)
Staining of cells + flow cytometry analysis

Ratio = $MFI_{INF}/MFI_{MOCK}$

| Figure | Cell permeabilization with Triton | SFV staining |
|---|---|---|
| 8A | No | MoAb anti-SU |
| 8B | No | Plasma from SFV-infected and uninfected individuals |

## S2D Fig. Schematic description of experiments presented in Fig 9A and 9B

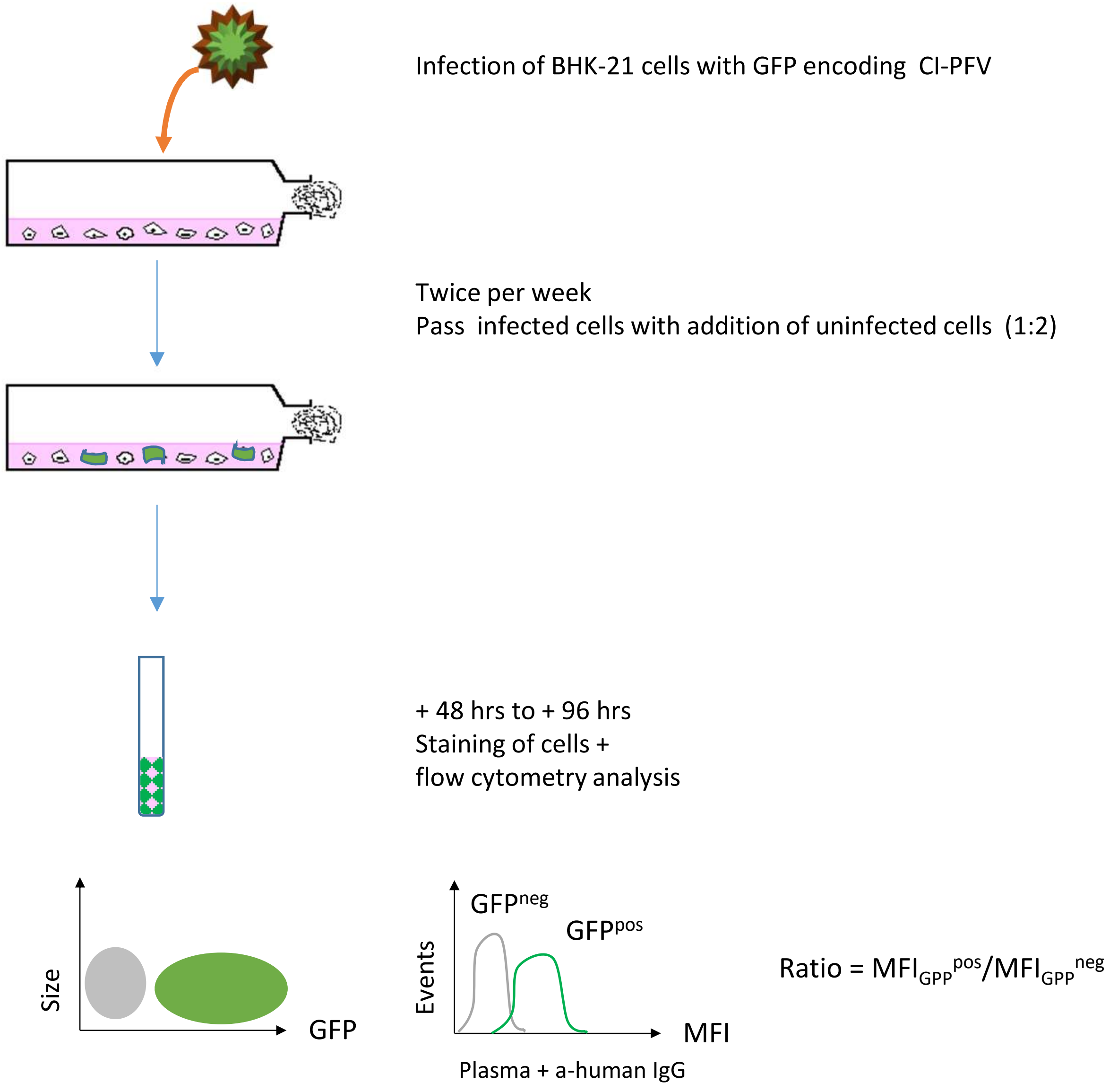


| Figure | Cell permeabilization with Triton | SFV staining |
|---|---|---|
| 9A-B | No | Plasma from SFV-infected and uninfected individuals |

## S2E Fig. Schematic description of experiments presented in Fig 9C and 9D

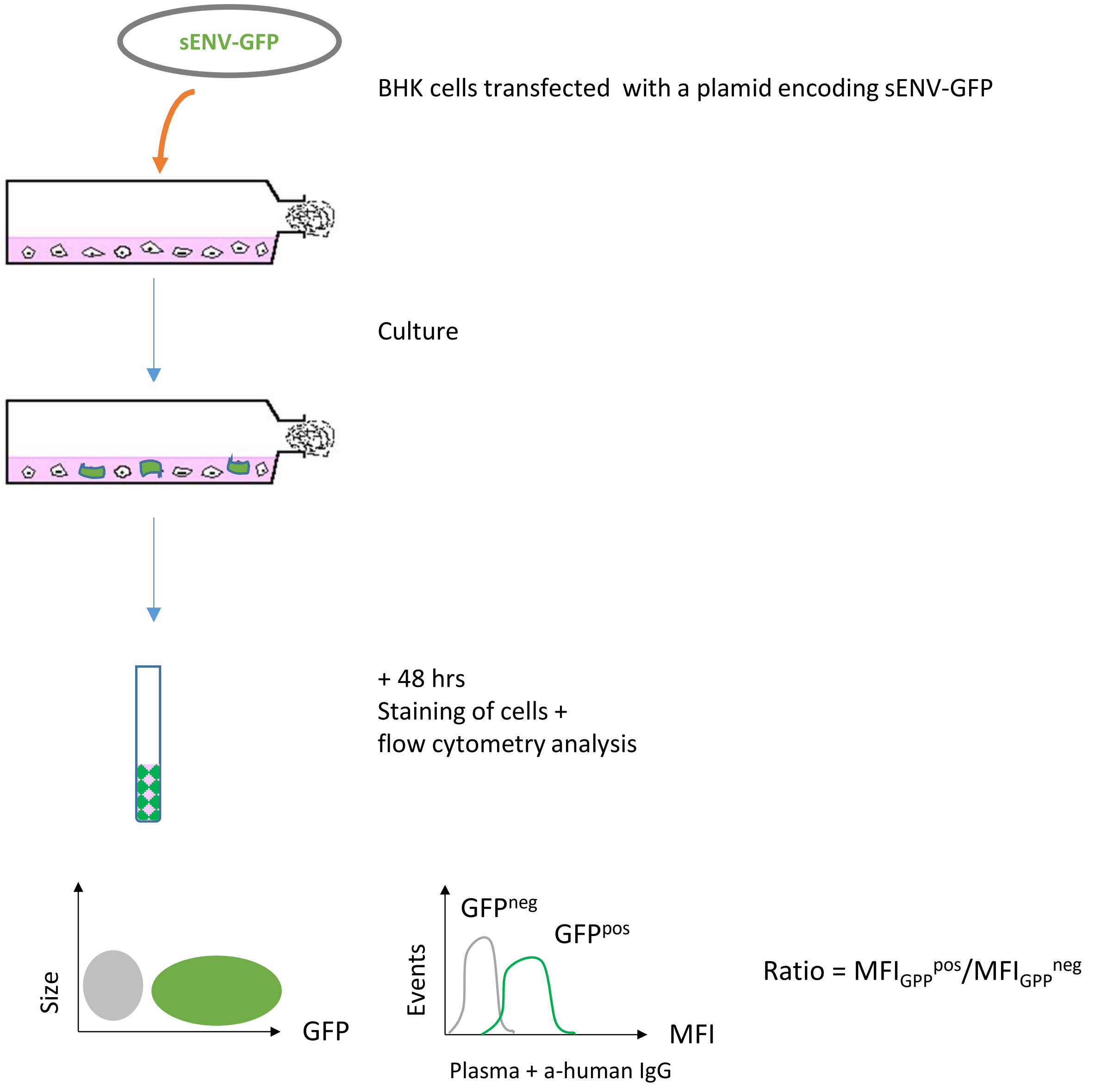


| Figure | Cell permeabilization with Triton | SFV staining |
|---|---|---|
| 9C-D | No | Plasma from SFV-infected and uninfected individuals |

**S2F Fig. Schematic description of experiments presented in Fig 10, 11, S8-10 and S1 to S4 movies**

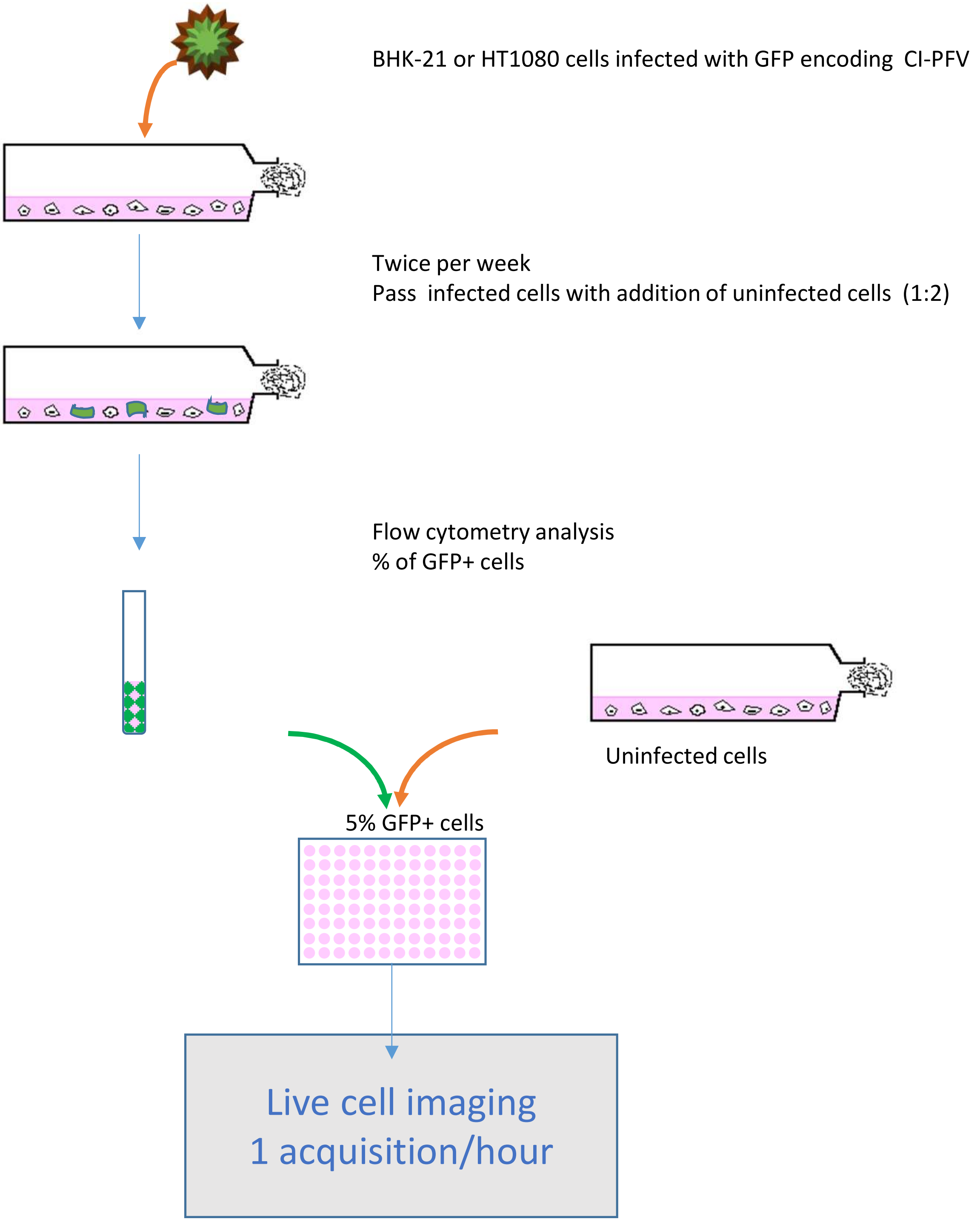


| Output | Objective |
|---|---|
| S1 to S4 Movies | x4 |
| Fig 10, 11, S8-S10 | X20 |
